# Supplementary material for: A Multimodal Educational Boot Camp for Training Fellows in Pediatric Extracorporeal Membrane Oxygenation (ECMO)
Source: MedEdPORTAL. 2024 Oct 17;20:11455. doi: 10.15766/mep_2374-8265.11455 (PMC11485016; doi:10.15766/mep_2374-8265.11455)
Supplement: Supplementary file 1 — Pneumothorax Simulation Case.docxECMO Pump Failure Simulation Case.docxCircuit Pressures Chart.docxTabletop ECMO Puzzle.pdfSample Agenda.docxIntroduction to ECMO.pptxECMO Knowledge Quiz.docxCircuit Components - Blank.pdfCircuit Components - Answers.docxCircuit Pressures Chart - Answers.docxPostsurvey.docx [file mep_2374-8265.11455-s001.zip › A. Pneumothorax Simulation Case.docx]

| **Appendix A**    **SIMULATION CASE TITLE: VA ECMO / tension pneumothorax**    **AUTHORS: Brian Gray, Kamal Abulebda, Nathan Swinger**    **LEARNER AUDIENCE: clinical fellows** | |
| --- | --- |
| **PATIENT NAME: John Parlow**    **PATIENT AGE: 2 years old**    **CHIEF COMPLAINT:** two-year-old child with viral myocarditis on VA-ECMO    **PHYSICAL SETTING: patient room in pediatric intensive care unit** | |
|  | |
| **Brief Narrative Description of Case** | This is a two-year-old child with viral myocarditis. 2 hours ago, he was cannulated onto VA ECMO during CPR with 16 Fr venous and 12 Fr arterial cannulas. *The team is called to evaluate the patient at bedside due to hypotension and low access pressures on the ECMO circuit.* Patient is currently on Epinephrine drip at 0.02 mcg/kg/min, and milrinone drip at 0.5 mcg/kg/min. |
| **Primary Learning Objectives** | - Recognize low access pressures limiting ECMO circuit flow - Display knowledge of common methods to improve low access pressures - Verbalize thought process/differential as common therapies fail to improve low access pressures - Enacts appropriate patient resuscitative therapies - Request chest radiograph and echocardiogram to evaluate pneumothorax, tamponade, and catheter malposition - Return patient to ECMO circuit in a timely and safe fashion |
| **Critical Actions** | - Identify low access pressures and inadequate ECMO flow - Notify ECMO technician - Administer fluid bolus (20mL/kg of 0.9 saline) and increase pressor for hypotension - Diagnose pneumothorax on chest radiograph and order echocardiogram - Ask for supplies to place right chest tube vs. needle decompression |
| **Learner Preparation or Prework** | *Introduction to ECMO circuit components including how to assess access pressures on ECMO circuit (addressed during the large group session attended by all boot camp participants prior to small group activity rotations on identification of ECMO circuit components)* |

ROOM SET UP AND EQUIPMENT:

Clinical Setting: ICU bedside for a patient on ECMO

Patient Set Up:

Mechanical vent with settings: PIP 20, PEEP 10, Rate 10, PS 10

16 Fr Venous ECMO cannula and 12 Fr Arterial ECMO Cannula in R neck

Femoral Central Venous Catheter

Radial Arterial Line

ETT

ECMO Display Monitor with flows and ECMO pressures

| **Supplies** | **Medication and Fluids** | **Other Supplies** |
| --- | --- | --- |
| ECMO Circuit / ECMO Simulator | Crystalloid and/or 5 % albumin bags | Mannequin |
| Patient bed | RBCs/ Platelets | Code Sheet |
| Backboard for chest compression | Epinephrine, Atropine, Calcium, Sodium Bicarbonate code doses | Lab and Xray results available to hand over to team |
| Ventilator | Center specific anticoagulation drip | 2 inch 18 gauge angiocath |
| IV Pump | Center specific sedation drips |  |
| Bedside ECMO Cart |  |  |
| Ventilation Bag - self inflating or anesthesia bag based on institutional preference |  |  |
| Suction |  |  |
| Stethoscope |  |  |

| **INITIAL PRESENTATION** | | | |
| --- | --- | --- | --- |
| **Initial Vital Signs** | HR: 150, BP: 68/38(48), CVP: 14, SpO2: 88%, SvO2: 30%  Venous Pressure: -90  Pre-Oxy Pressure: 130  Bladder Pressure: -88  Post Oxy Pressure: 125 | | |
| **Overall Setting and Appearance** | Participants encounter a child on VA-ECMO support in the pediatric ICU. The child is intubated and sedated. This scenario takes place in an actual pediatric ICU room or an identical space. | | |
| **Standardized Participants (and Their Roles in the Room at Case Start)** | 1. ECMO tech or clinician - embedded participant in early stages  This standardized participant is encouraged to function based on their actual role in a clinical scenario. They were given the scenario script ahead of time but were expected to practice within their scope of practice as an ECMO clinical specialist.    2. Bedside RN - embedded participant in early stages  Similar to above, this standardized participant is encouraged to function based on their actual role in a clinical scenario. They were given the scenario script ahead of time but were expected to practice within their scope of practice as a bedside neonatal ICU nurse.    3. Surgeon on standby if needed | | |
| **HPI** | This is a two-year-old child with viral myocarditis. 2 hours ago, he was cannulated onto VA ECMO during CPR with 16 Fr venous and 12 Fr arterial cannulas. *The team is called to evaluate the patient at bedside due to hypotension and low access pressures on the ECMO circuit.* | | |
| **Past Medical/Surgical History** | **Medications** | **Allergies** | **Family History** |
| none | Patient is currently on Epinephrine drip at 0.02 mcg/kg/min, and milrinone drip at 0.5 mcg/kg/min. | none | non-contributory |
| **Physical Examination** | | | |
| **General** | Intubated, sedated | | |
| **HEENT** | Eyes closed | | |
| **Neck** | ECMO cannulae in place | | |
| **Lungs** | **No breath sounds on the right, asymmetric chest rise** | | |
| **Cardiovascular** | **Tachycardia, ECMO flow murmur heard, very weak central pulses, no palpable peripheral pulses, capillary refill time 5 sec** | | |
| **Abdomen** | Abdomen mildly distended but soft | | |
| **Neurological** | Deeply sedated, no spontaneous movement | | |
| **Skin** | Dry, intact | | |
| **GU** | No abnormalities | | |
| **Psychiatric** | N/A | | |

**Labs/Diagnostic Results:**

CXR: **right-sided tension pneumothorax**, cannula in appropriate position

ABG: pH 7.11/pCO2 45 mmHg/pO2 125 mmHg/BD -10, Lactate 4.1 mmol/L

BMP: Na 137 mmol/L, K 3.5 mmol/L, Cl 105 mmol/L, CO2 16 mmol/L, BUN 18 mg/dL, Cr 0.7 mg/dL

Mg 2.5 mg/dL, Phos 5 mg/dL

LFT: ALT 132 Units/L, AST 243 Units/L, ALK 130 Units/L

Anti Xa Level 0.1 IU/L

**INSTRUCTOR NOTES - CHANGES AND CASE BRANCH POINTS**


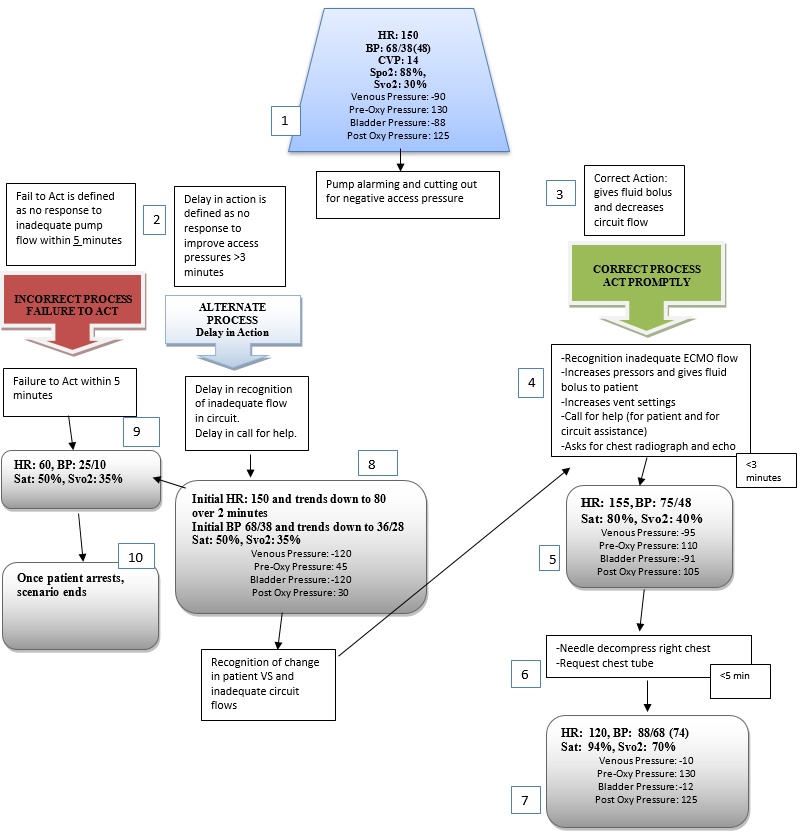


**Ideal Scenario Flow**

*The learners enter the patient’s room and are notified by the bedside nurse that the ECMO pump has been alarming due to negative access pressures. They immediately check all available monitors and find the patient to be hypotensive. The learners order a fluid bolus and increase the patient’s pressors to address the hypotension without significant improvement. Vent settings are increased for hypoxia. The patient’s physical exam is notable for absent breath sounds on the right chest. The learners then call for additional help from an ECMO clinician and ECMO technician, and in the meantime order a chest radiograph and echocardiogram. The CXR confirms the presence of a right-sided tension pneumothorax. The learners then communicate that they would like to proceed with either a chest tube placement or needle decompression, at which point the scenario ends.*

**Anticipated Management Mistakes**

1. *Failure to identify tension pneumothorax: We found it helpful to continue to trend down the blood pressure and oxygen saturation to encourage learners to continue to look for causes of hypotension. The bedside nurse can also prompt learners with “I’m not sure I heard breath sounds on both sides the last time I checked.”*
2. *Difficulty identifying patterns in ECMO circuit pressures: We incorporated an ECMO circuit pressure chart activity in our boot camp to help learners become familiar with various changes in circuit pressures and flow associated with commonly occurring ECMO circuit problems.*

**Debrief Guide**

1. Ask participants how they felt the simulation went – what went well/what was challenging about the scenario
2. Review the main learning points from the scenario – discuss how the group came to the conclusion that the patient had a pneumothorax (circuit pressures, hypotension, physical exam), and what actions they took to treat it
3. Ask participants to comment on their communication with each other and the ECMO technician during the case. Did they feel stressed? How did that affect their performance?
