# Supplementary material for: A Multimodal Educational Boot Camp for Training Fellows in Pediatric Extracorporeal Membrane Oxygenation (ECMO)
Source: MedEdPORTAL. 2024 Oct 17;20:11455. doi: 10.15766/mep_2374-8265.11455 (PMC11485016; doi:10.15766/mep_2374-8265.11455)
Supplement: Supplementary file 1 — Pneumothorax Simulation Case.docxECMO Pump Failure Simulation Case.docxCircuit Pressures Chart.docxTabletop ECMO Puzzle.pdfSample Agenda.docxIntroduction to ECMO.pptxECMO Knowledge Quiz.docxCircuit Components - Blank.pdfCircuit Components - Answers.docxCircuit Pressures Chart - Answers.docxPostsurvey.docx [file mep_2374-8265.11455-s001.zip › B. ECMO Pump Failure Simulation Case.docx]

| **Appendix B**    **SIMULATION CASE TITLE: VA ECMO / Pump Failure**    **AUTHORS: Brian Gray, Kamal Abulebda, Nathan Swinger**    **LEARNER AUDIENCE: clinical fellows** | |
| --- | --- |
| **PATIENT NAME: Peter Fowler**    **PATIENT AGE: 4 weeks old**    **CHIEF COMPLAINT: on day 2 of VA-ECMO for pneumonia with myocarditis**    **PHYSICAL SETTING: patient room in neonatal intensive care unit** | |
|  | |
| **Brief Narrative Description of Case** | *The patient is a 4-week-old male with pneumonia and myocarditis who is on day 2 of VA-ECMO. The team is called to evaluate the patient at bedside due to hypotension and hypoxia. Overall learner goals are to recognize pump failure, troubleshoot the ECMO circuit, and demonstrate effective communication with team members.* |
| **Primary Learning Objectives** | - *Recognize pump failure based on ECMO circuit pressure values* - *Express knowledge of corrective steps to take during pump failure, including performing a hand crank if pump reset fails* - *Demonstrate effective communication with team members* - *Call for help early upon recognition of pump failure* |
| **Critical Actions** | - *Immediate recognition of ECMO circuit pressure changes* - *Identify pump malfunction* - *Call ECMO clinician for help* - *Check both venous and arterial limb of circuit* - *Attempt to restart pump* - *Manually hand crank pump when it fails to restart* |
| **Learner Preparation or Prework** | *Introduction to ECMO circuit components including how to manually hand crank in cases of pump failure (addressed during the large group session attended by all boot camp participants prior to small group activity rotations on identification of ECMO circuit components)* |

ROOM SET UP AND EQUIPMENT:

A chair and computer/workstation for an ECMO tech is needed. Eigenflow (ECMO simulator) should be set up behind the ECMO circuit so that its tubing is less visible. The ventilator should be placed on the patient’s left side with the IV pole behind it. The ECMO circuit should be on the patient’s right side.

| **Supplies** | **Medication and Fluids** |
| --- | --- |
| ECMO Circuit / Eigenflow ECMO simulator | NS and 5% albumin bags |
| Patient bed | RBCs/ Plts |
| Backboard for chest compression | Epi, Atropine, Calcium, NaHco3 code doses |
| Ventilator |  |
| Mannequin |  |

**Initial Room Set Up**

| **MONITOR DISPLAY** | | | | **ACCESS** | | | | | | | **Special Notes:** |
| --- | --- | --- | --- | --- | --- | --- | --- | --- | --- | --- | --- |
| X | **ECG** | **130** | **BP 68/38** | **X** | **Fem CVC double lumens/UVC** | | 4 Fr femoral cath at 5 cm | |  | |  |
| X | **Sa02** | **97%** | **ART line BP** | **X** | **Radial A line** with 2.5 NS | | | |  |  |  |
| X | **RR** | **10** |  | X | **ECMO cannula** | | | | | |  |
| **INTRAVENOUS FLUIDS** | | | | | | | | | | | |
| **X** | **IV fluids:  D10 ½ NS** | | | | | **Rate:  20 ml/h  (Fem CVC)** | | | | | |
| **MEDICATIONS** | | | | | | | | | | | |
| **Ampicillin** | | | | | | **Heparin (circuit side)** | | | | | |
| **Gentamycin** | | | | | | **Fentanyl 2 mcg/kg/hr (7cmg/hr) (Fem CVC)** | | | | | |
| **Epinephrine 0.2 mcg/kg (Fem CVC)** | | | | | |  | | | | | |
| **OXYGEN SUPPLIES AT BEDSIDE** | | | | | | | | | | | |
| X | **Self-inflating Bag/Mask** | | | X | **End tidal CO2** | | | x | | **Wall Oxygen/Suction with tubing** | |
| **X** | **Mechanical vent with settings (PICU): PIP 20, PEEP 10, Rate 10, PS 10** | | | | | | |  | |  | |
| AIRWAY MANAGEMENT | | | | | | | | | | | |
| **X** | **Laryngoscope:** | | | **Blade sizes**:  0, 1 Miller | | | | | | | |
| **X** | **ETT:** | | | **Sizes**: 3.5-4.0 taped at 10 cm | | | | | | | |
| **CARTS AND EQUIPMENT** | | | | | | | | | | | |
| **X** | Airway tote | | | X | Stethoscope | | | X | | Radiant warmer/Patient bed | |
|  | Bulb syringe | | | X | Code cart | | | X | | Replogle | |
|  | Defibrillator | | | X | Portable I-stat | | | X | | IV pump/pole | |
| MISC ITEMS | | | | | | | | | | | |
| X | **Vocera phone** | | | X | **Lab/Diagnostic Results** | | |  | |  | |

| **INITIAL PRESENTATION** | | | |
| --- | --- | --- | --- |
| **Initial Vital Signs** | HR: 180, BP: 50/20, SpO2: 75%, SvO2: 75%  P1: 20, P2: 25, P3: 20, P4: 20 | | |
| **Overall Setting and Appearance** | Participants encounter an infant on VA-ECMO support in the Neonatal ICU. The child is intubated and sedated. The mannequin appears stable and is not moving. This scenario takes place in an actual neonatal ICU room or an identical space. | | |
| **Standardized Participants (and Their Roles in the Room at Case Start)** | 1. ECMO tech or clinician - embedded participant in early stages  This standardized participant is encouraged to function based on their actual role in a clinical scenario. They were given the scenario ahead of time but were expected to practice within their scope of practice as an ECMO clinical specialist.    2. Bedside RN - embedded participant in early stages  Similar to above, this standardized participant is encouraged to function based on their actual role in a clinical scenario. They were given the scenario ahead of time but were expected to practice within their scope of practice as a bedside neonatal ICU nurse.    3. Surgeon on standby if needed | | |
| **HPI** | 4 week old male with pneumonia and myocarditis. Patient was placed on VA ECMO 2 days ago secondary to refractory cardiogenic shock. Patient is currently on Epinephrine drip at 0.02 mcg/kg/min, and milrinone drip at 0.5 mcg/kg/min. | | |
| **Past Medical/Surgical History** | **Medications** | **Allergies** | **Family History** |
| none | Epi drip  Milrinone drip | none | non-contributory |
| **Physical Examination** | | | |
| **General** | Sedated, on vent | | |
| **HEENT** | Eyes closed | | |
| **Neck** | ECMO cannulae in place | | |
| **Lungs** | Breath sounds equal bilaterally | | |
| **Cardiovascular** | RRR, **very weak central pulses, no palpable peripheral pulses, capillary refill 3 seconds** | | |
| **Abdomen** | Mildly distended but soft | | |
| **Neurological** | Deeply sedated, no spontaneous movements | | |
| **Skin** | Intact, dry | | |
| **GU** | No abnormalities | | |
| **Psychiatric** | N/A | | |

**Labs/Diagnostic Results:**

CXR: No major findings, cannula in appropriate position

ABG: pH 7.38/pCO2 45mmHg/pO2 125mmHg/BE +2, Lactate 1.1 mmol/L

BMP: Na 137 mmol/L, K 3.5 mmol/L, Cl 105 mmol/L, CO2 22 mmol/L, BUN 18 mg/dL, Cr 0.3 mg/dL

Mg 2.5 mg/dL, Phos 5 mg/dL

LFT: ALT 22 Units/L, AST 33 Units/L, ALK 130 Units/L

ACT: 180 s, Anti Xa Level 0.55 IU/mL

**INSTRUCTOR NOTES - CHANGES AND CASE BRANCH POINTS**

THE BELOW ALGORITHM WAS UTILIZED BY THE INSTRUCTOR TO ADVANCE THE CASE BASED ON PARTICIPANT ACTIONS.

**
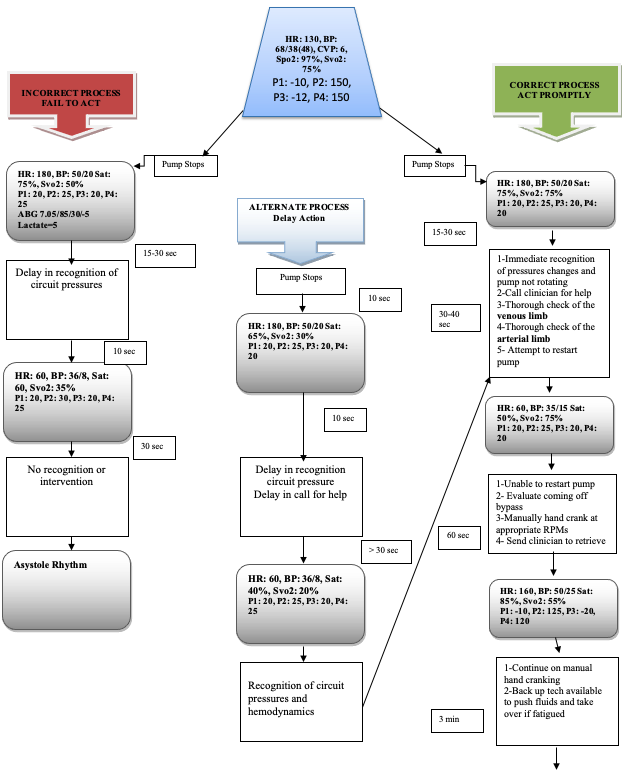
**

**INSTRUCTOR NOTES - CHANGES AND CASE BRANCH POINTS** (CONTINUED)


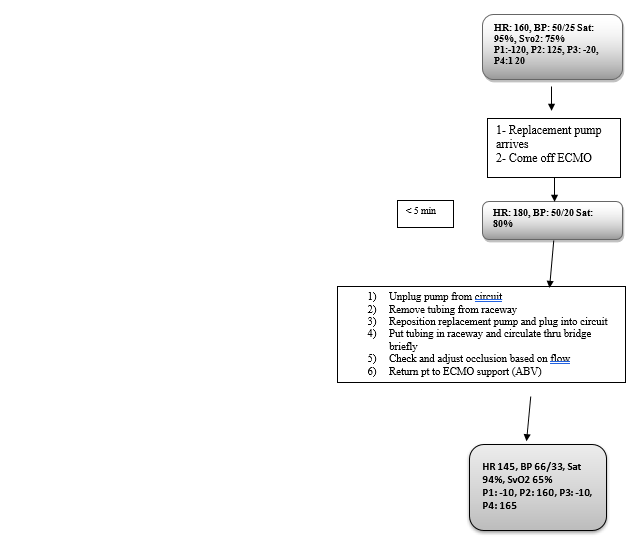


**Ideal Scenario Flow**

*The learners enter the patient room and are notified by the bedside nurse that an alarm is going off on the ECMO circuit and that the patient has become more hypoxic and hypotensive. They immediately check the patient’s monitors and recognize the abnormal ECMO circuit pressures, and that the pump is not rotating. They call for assistance from an ECMO clinician and ECMO technician as soon as possible. While waiting for additional help to arrive, they thoroughly assess both the venous and arterial limbs of the circuit and try to restart the pump. When they are unable to accomplish this, they start to manually hand crank the pump and call for a replacement pump to be delivered to the patient room. They continue cranking until the new pump and additional ECMO personnel arrive and work to stabilize the patient. Scenario ends with installation of new pump and improvement in patient vitals and circuit pressures.*

**Anticipated Management Mistakes**

1. *Delay in recognition of circuit pressure derangements: We found it helpful to allow the blood pressure and oxygen saturation to continue to drop to signal to the learners to keep looking for a problem*
2. *Failure to start manually hand cranking the ECMO pump while waiting for a replacement: the manual hand crank was specifically addressed during the large group introductory session demonstrating the ECMO circuit components.*

**Debrief Guide**

1. Ask participants how they felt the simulation went – what went well/what was challenging about the scenario
2. Review the main learning points from the scenario – what clinical findings helped them realize there was pump failure, and what actions did they take to continue to support the patient while waiting for a replacement
3. Ask participants to comment on their communication with each other and the ECMO technician during the case. Did they feel stressed? How did that affect their performance?
