# Supplementary material for: A Multimodal Educational Boot Camp for Training Fellows in Pediatric Extracorporeal Membrane Oxygenation (ECMO)
Source: MedEdPORTAL. 2024 Oct 17;20:11455. doi: 10.15766/mep_2374-8265.11455 (PMC11485016; doi:10.15766/mep_2374-8265.11455)
Supplement: Supplementary file 1 — Pneumothorax Simulation Case.docxECMO Pump Failure Simulation Case.docxCircuit Pressures Chart.docxTabletop ECMO Puzzle.pdfSample Agenda.docxIntroduction to ECMO.pptxECMO Knowledge Quiz.docxCircuit Components - Blank.pdfCircuit Components - Answers.docxCircuit Pressures Chart - Answers.docxPostsurvey.docx [file mep_2374-8265.11455-s001.zip › C. Circuit Pressures Chart.docx]

ECMO Circuit Pressures Chart

|  | **Venous Pressure** | **Pre-Membrane Pressure** | **Post-Membrane Pressure** | **Delta Pressure (Transmembrane)** | **Flow** |
| --- | --- | --- | --- | --- | --- |
| **Hypovolemia** |  |  |  |  |  |
| **Tension Pneumothorax** |  |  |  |  |  |
| **Cardiac Tamponade** |  |  |  |  |  |
| **Increased Systemic Vascular Resistance** |  |  |  |  |  |
| **Oxygenator/Membrane Failure** |  |  |  |  |  |
| **Small Arterial Cannula** |  |  |  |  |  |
| **Malpositioned Venous Cannula** |  |  |  |  |  |
