# Supplementary material for: A Multimodal Educational Boot Camp for Training Fellows in Pediatric Extracorporeal Membrane Oxygenation (ECMO)
Source: MedEdPORTAL. 2024 Oct 17;20:11455. doi: 10.15766/mep_2374-8265.11455 (PMC11485016; doi:10.15766/mep_2374-8265.11455)

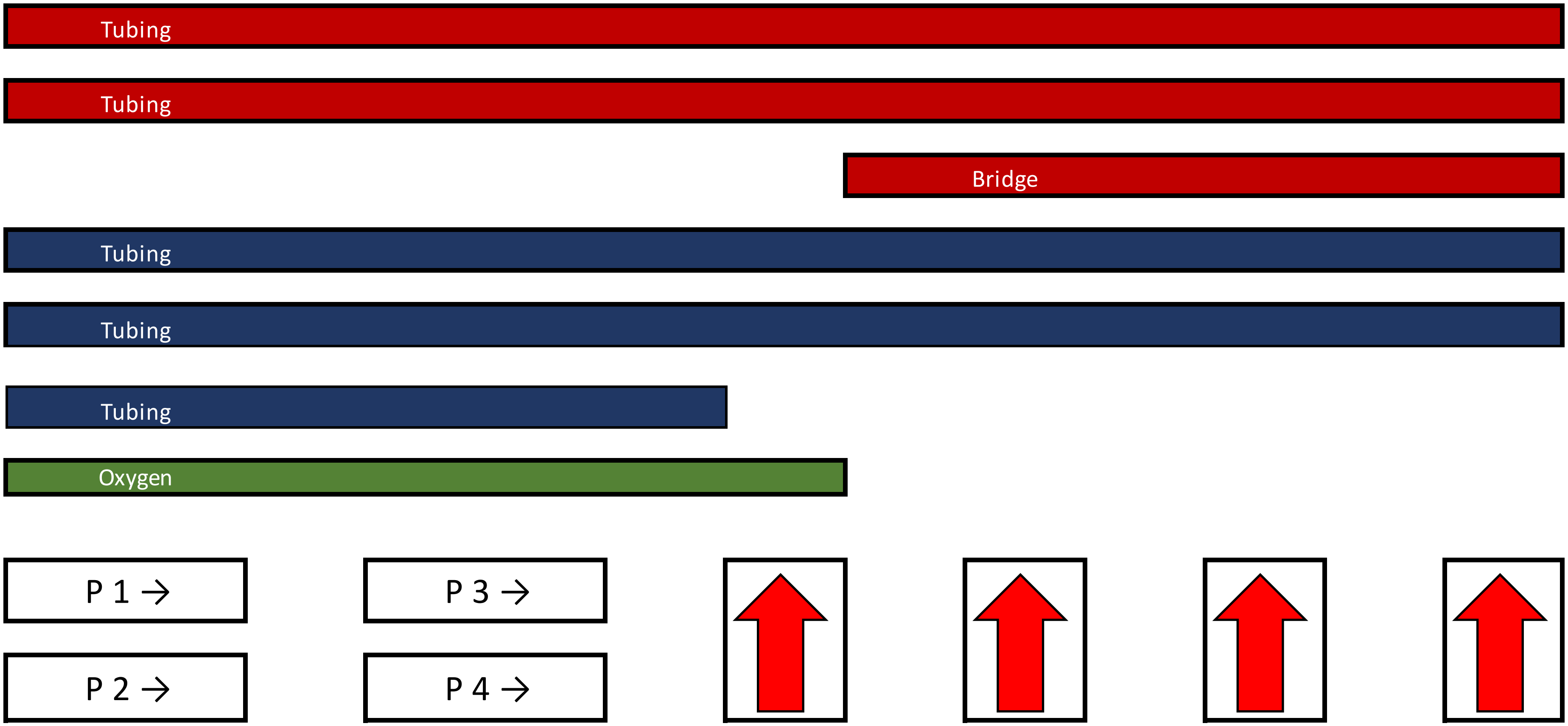

Recommended Activity Duration: 30 min

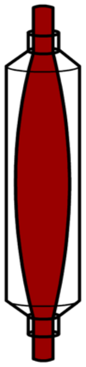

Bladder

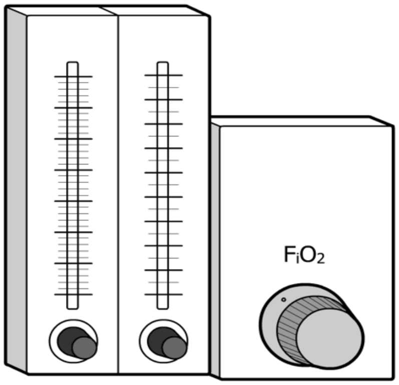

Sweep / Blender

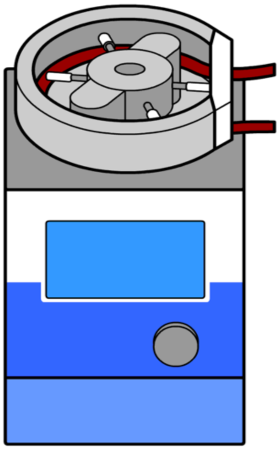

Roller Pump

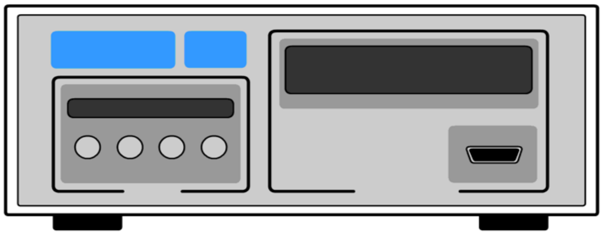

Ultrasonic Flow Monitor

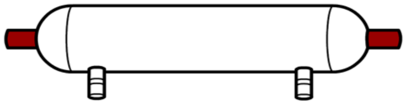

Heat Exchanger

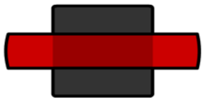

Bubble Detector

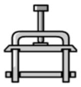

Clamp

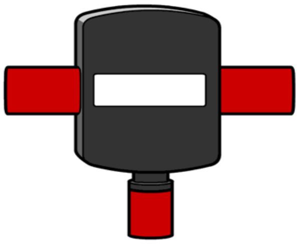

M2 Sensor

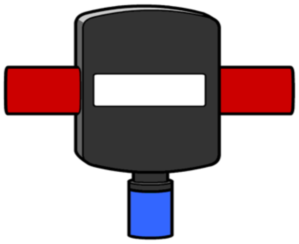

M2 Sensor

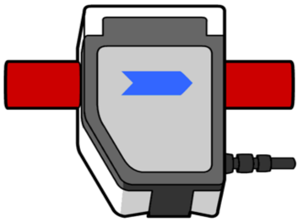

Ultrasonic Sensor

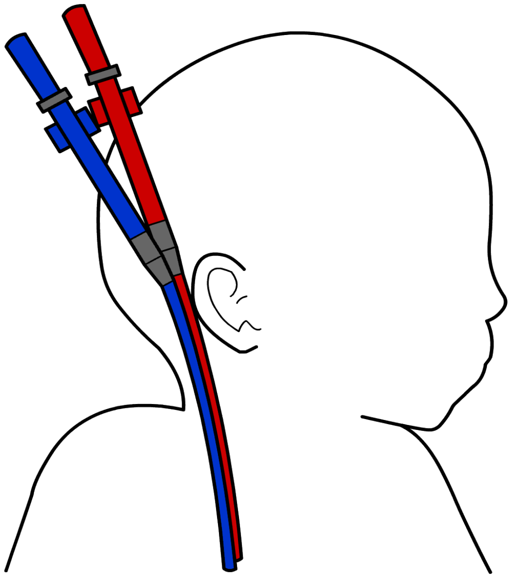

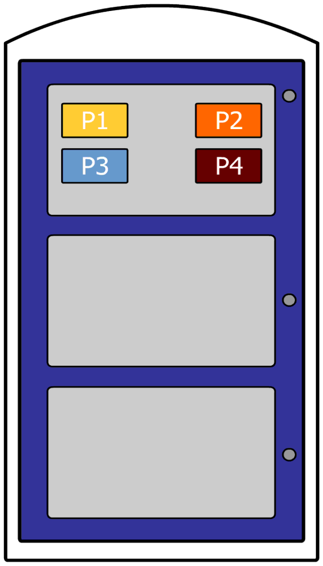

Pressure Monitor

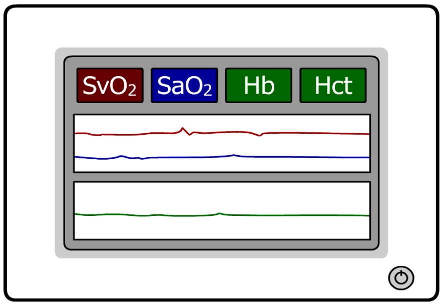

M2 Monitor

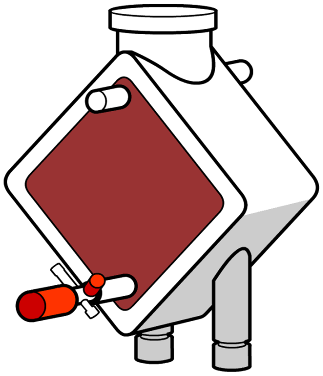

Oxygenator

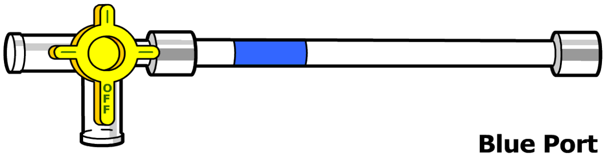

Blue Port

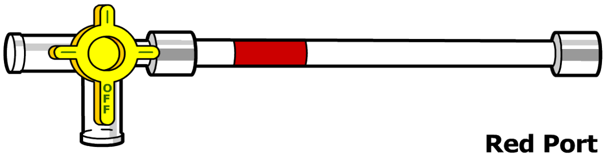

Red Port

Sample Answer Key for Tabletop Puzzle

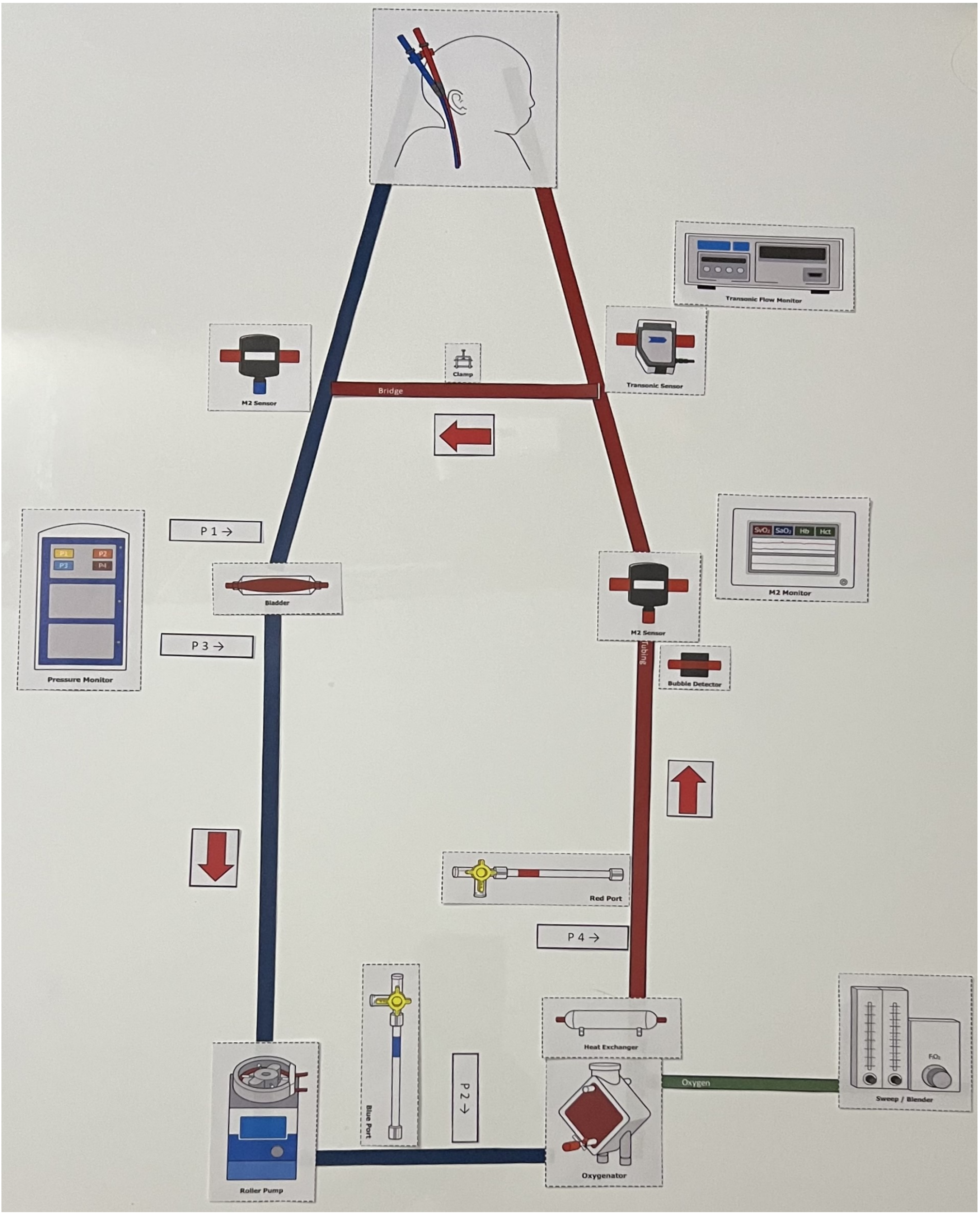

Supplement: Supplementary file 1 — Pneumothorax Simulation Case.docxECMO Pump Failure Simulation Case.docxCircuit Pressures Chart.docxTabletop ECMO Puzzle.pdfSample Agenda.docxIntroduction to ECMO.pptxECMO Knowledge Quiz.docxCircuit Components - Blank.pdfCircuit Components - Answers.docxCircuit Pressures Chart - Answers.docxPostsurvey.docx [file mep_2374-8265.11455-s001.zip › D. Tabletop ECMO Puzzle.pdf]
