# Supplementary material for: A Multimodal Educational Boot Camp for Training Fellows in Pediatric Extracorporeal Membrane Oxygenation (ECMO)
Source: MedEdPORTAL. 2024 Oct 17;20:11455. doi: 10.15766/mep_2374-8265.11455 (PMC11485016; doi:10.15766/mep_2374-8265.11455)
Supplement: Supplementary file 1 — Pneumothorax Simulation Case.docxECMO Pump Failure Simulation Case.docxCircuit Pressures Chart.docxTabletop ECMO Puzzle.pdfSample Agenda.docxIntroduction to ECMO.pptxECMO Knowledge Quiz.docxCircuit Components - Blank.pdfCircuit Components - Answers.docxCircuit Pressures Chart - Answers.docxPostsurvey.docx [file mep_2374-8265.11455-s001.zip › E. Sample Agenda.docx]

**ECMO Boot Camp for Fellows: Sample Agenda**

| **Large Group** | |
| --- | --- |
| Welcome/Lunch – introduction pptx, objectives and introduction of boot camp instructors | 45 min |
| Knowledge Pre-test MCQ | 15 min |
| Circuit Components Introduction with worksheet | 30 min |
| Break/Transition into Small Groups | 30 min |
| **Small Group Activity Stations:** small groups rotate through 4 stations, **30 minutes per station** + **5 minute transition period between stations** | 2 hrs 15 min |
| Guided Circuit Pressures Worksheet | |
| Simulation Scenario #1: pneumothorax | |
| Simulation Scenario #2: ECMO pump failure | |
| Tabletop Circuit Puzzle | |
| **Large Group:** Debrief, Post-test MCQ, feedback questionnaire | 45 min |
