# Supplementary material for: A Multimodal Educational Boot Camp for Training Fellows in Pediatric Extracorporeal Membrane Oxygenation (ECMO)
Source: MedEdPORTAL. 2024 Oct 17;20:11455. doi: 10.15766/mep_2374-8265.11455 (PMC11485016; doi:10.15766/mep_2374-8265.11455)
Supplement: Supplementary file 1 — Pneumothorax Simulation Case.docxECMO Pump Failure Simulation Case.docxCircuit Pressures Chart.docxTabletop ECMO Puzzle.pdfSample Agenda.docxIntroduction to ECMO.pptxECMO Knowledge Quiz.docxCircuit Components - Blank.pdfCircuit Components - Answers.docxCircuit Pressures Chart - Answers.docxPostsurvey.docx [file mep_2374-8265.11455-s001.zip › F. Introduction to ECMO.pptx]

## Slide 1
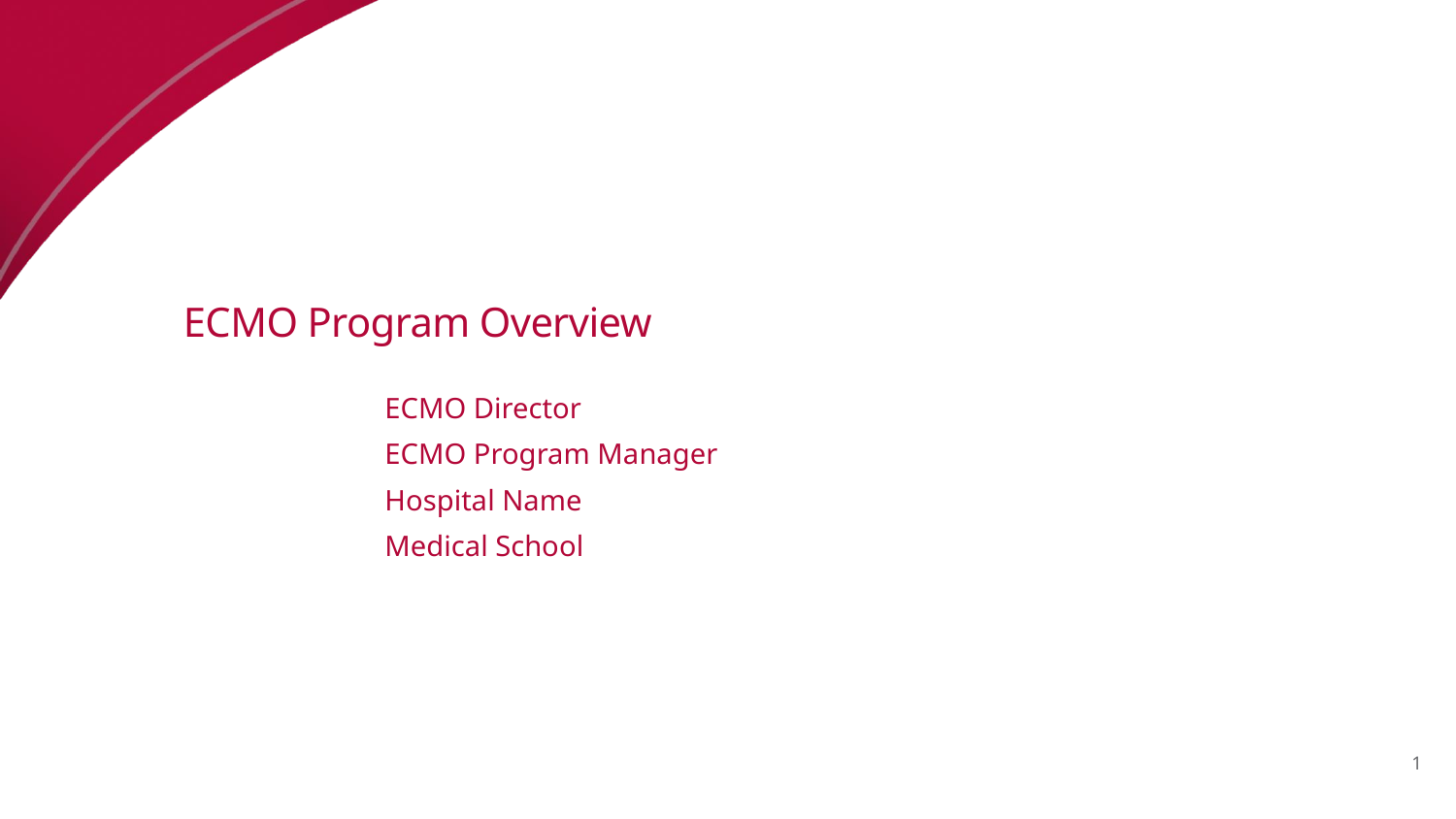

# ECMO Program Overview
ECMO Director
ECMO Program Manager
Hospital Name
Medical School
1

## Slide 2
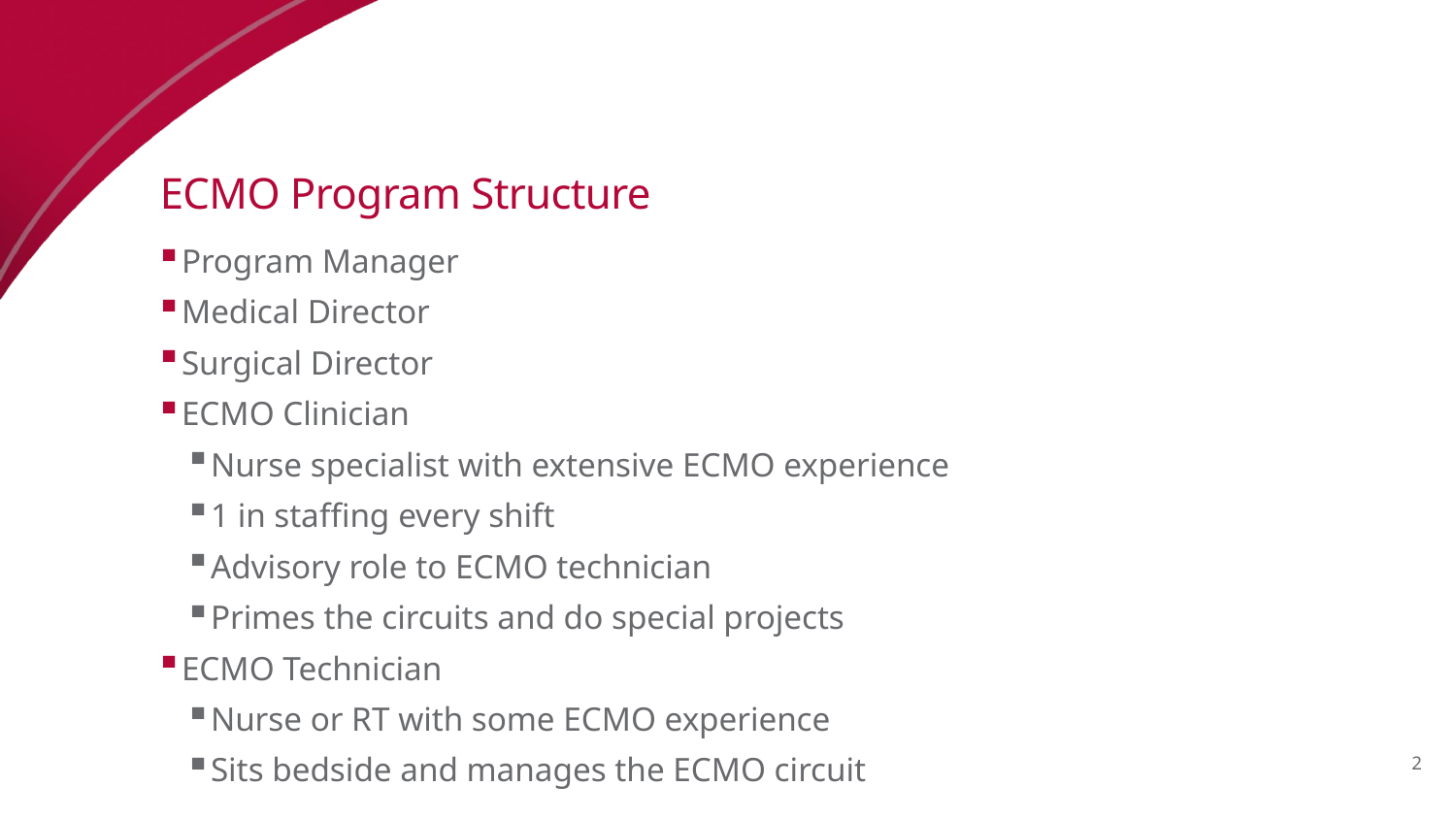

# ECMO Program Structure
Program Manager
Medical Director
Surgical Director
ECMO Clinician
Nurse specialist with extensive ECMO experience
1 in staffing every shift
Advisory role to ECMO technician
Primes the circuits and do special projects
ECMO Technician
Nurse or RT with some ECMO experience
Sits bedside and manages the ECMO circuit
2

## Slide 3
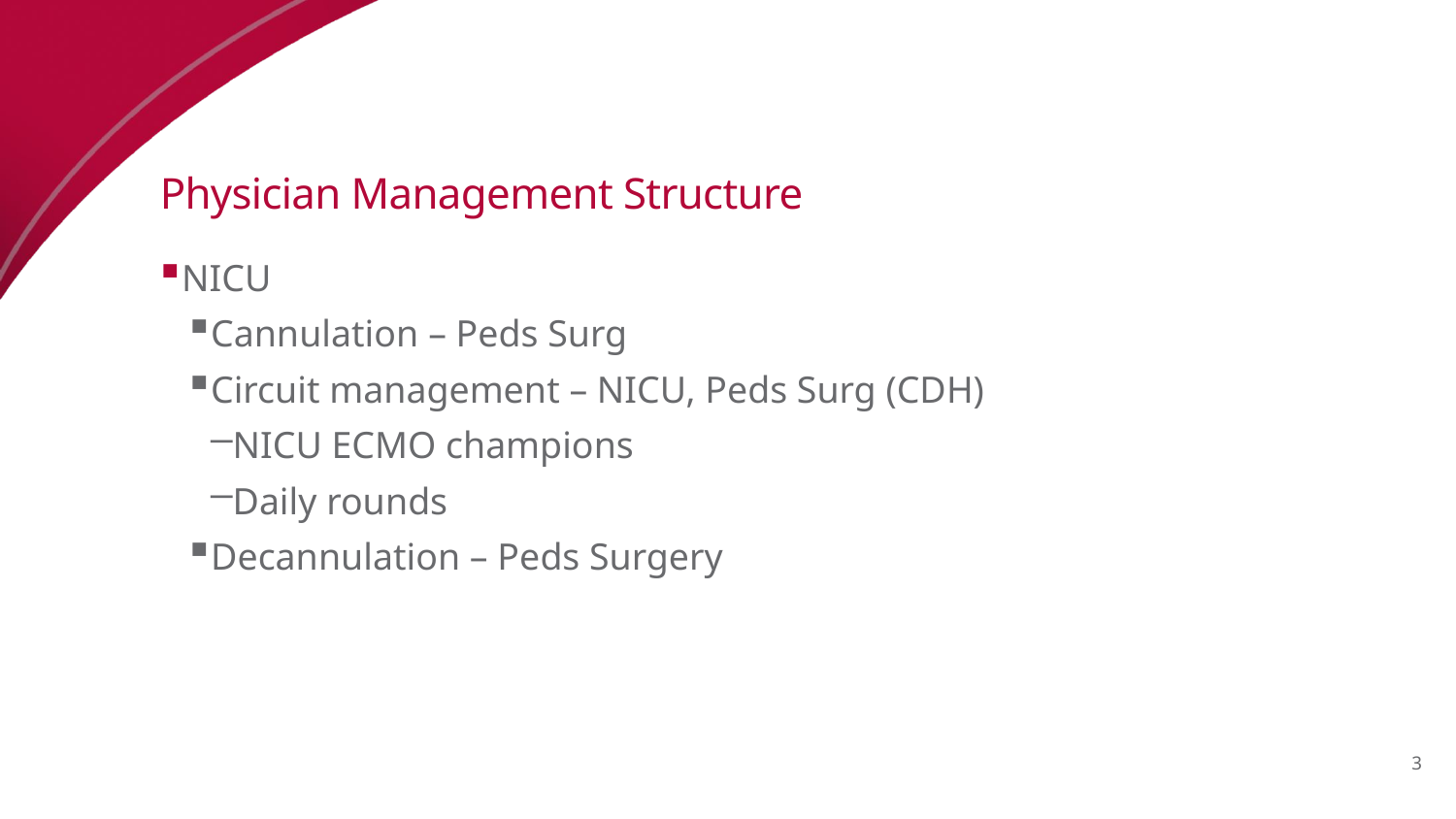

# Physician Management Structure
NICU
Cannulation – Peds Surg
Circuit management – NICU, Peds Surg (CDH)
NICU ECMO champions
Daily rounds
Decannulation – Peds Surgery
3

## Slide 4
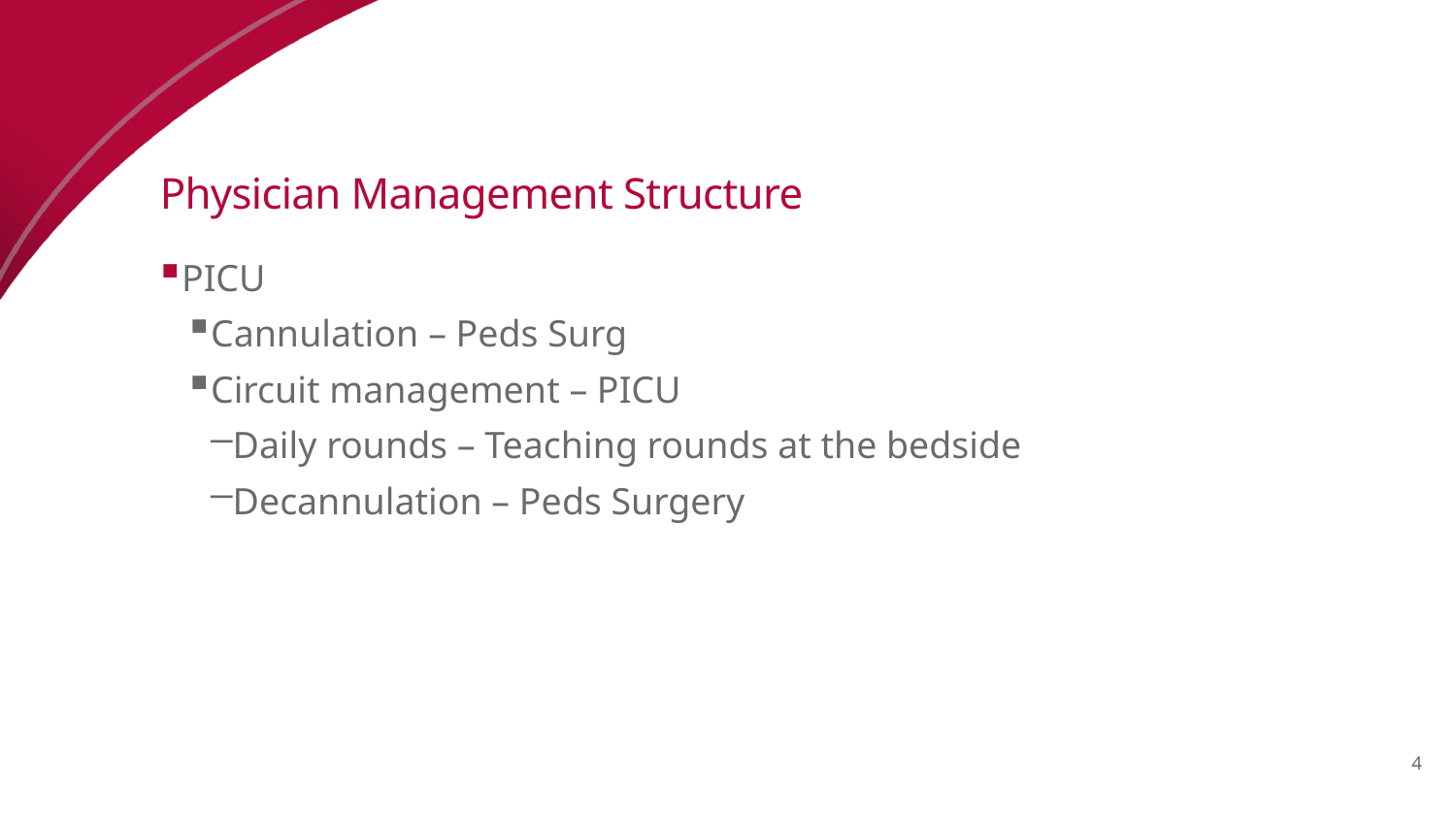

# Physician Management Structure
PICU
Cannulation – Peds Surg
Circuit management – PICU
Daily rounds – Teaching rounds at the bedside
Decannulation – Peds Surgery
4

## Slide 5
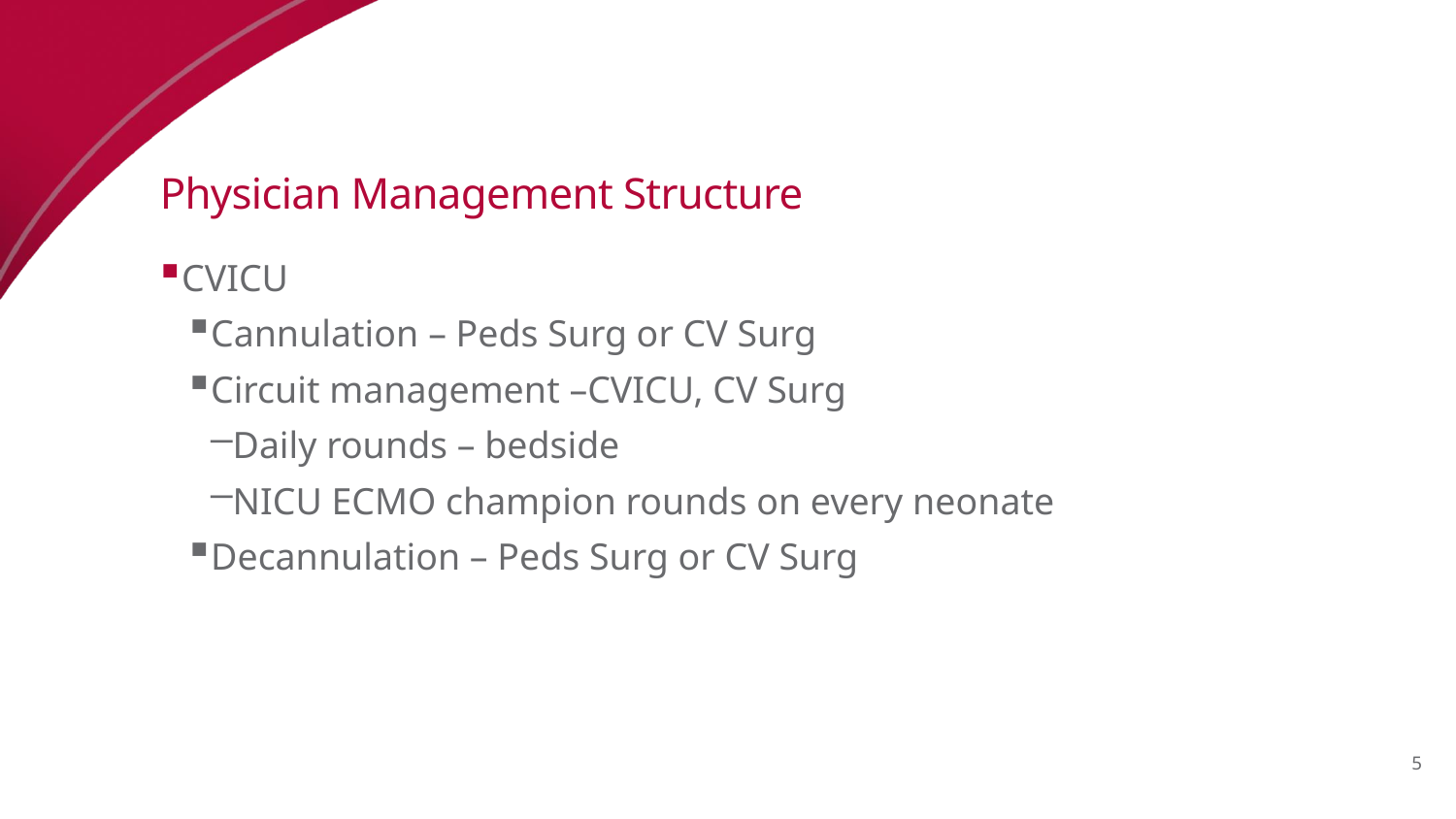

# Physician Management Structure
CVICU
Cannulation – Peds Surg or CV Surg
Circuit management –CVICU, CV Surg
Daily rounds – bedside
NICU ECMO champion rounds on every neonate
Decannulation – Peds Surg or CV Surg
5

## Slide 6
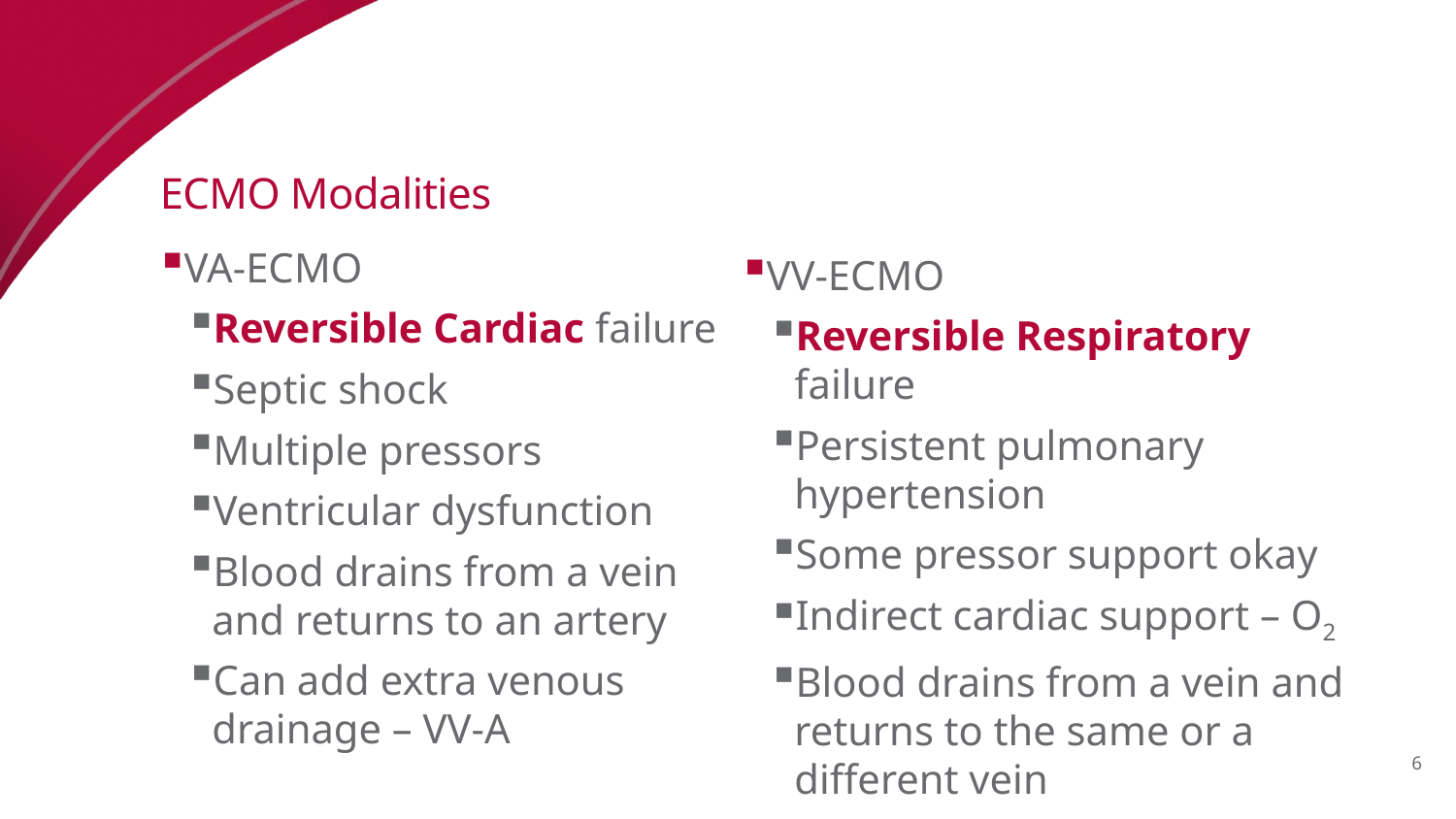

# ECMO Modalities
VA-ECMO
Reversible Cardiac failure
Septic shock
Multiple pressors
Ventricular dysfunction
Blood drains from a vein and returns to an artery
Can add extra venous drainage – VV-A
VV-ECMO
Reversible Respiratory failure
Persistent pulmonary hypertension
Some pressor support okay
Indirect cardiac support – O2
Blood drains from a vein and returns to the same or a different vein
6

## Slide 7
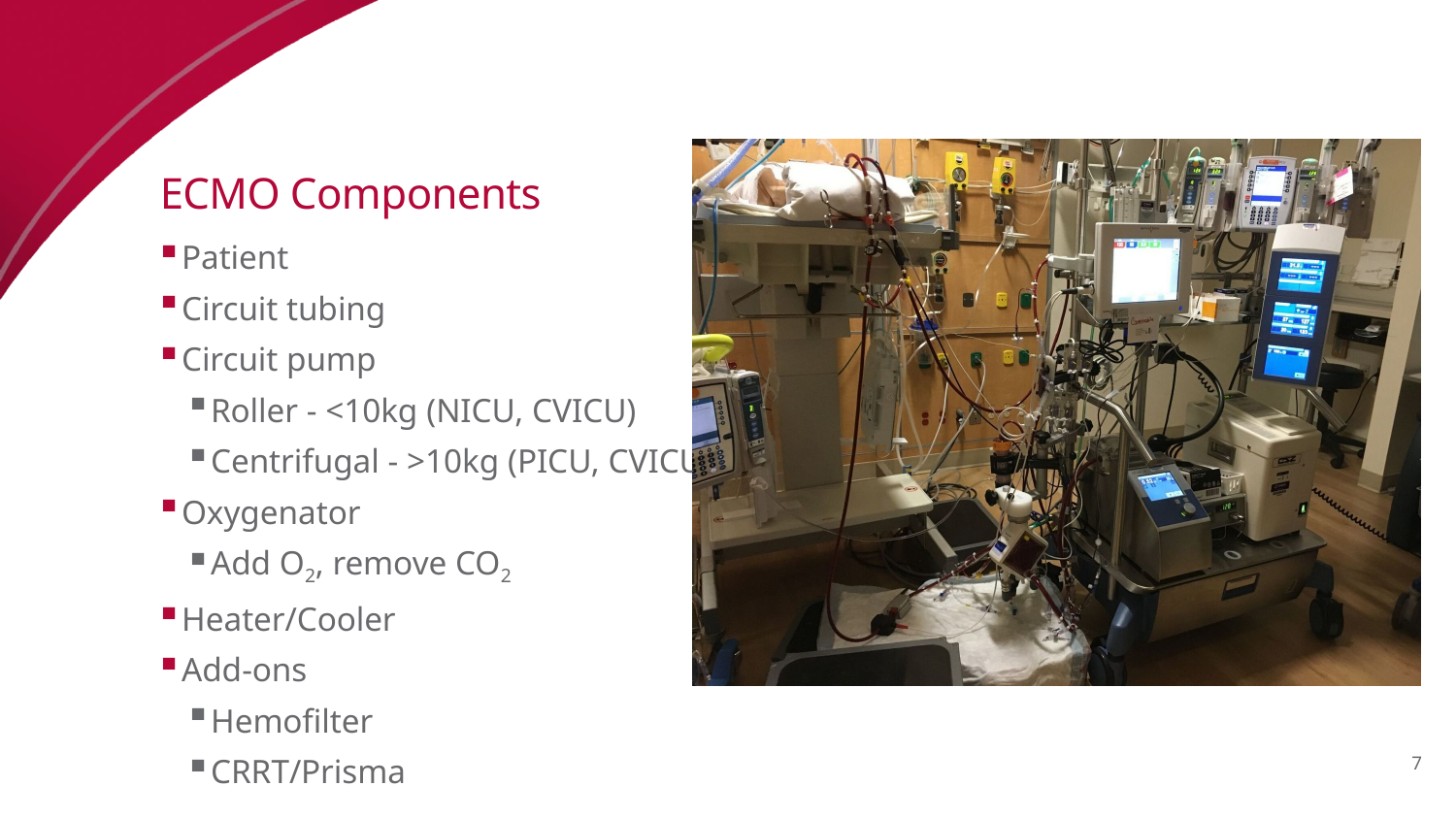

# ECMO Components
Patient
Circuit tubing
Circuit pump
Roller - <10kg (NICU, CVICU)
Centrifugal - >10kg (PICU, CVICU)
Oxygenator
Add O2, remove CO2
Heater/Cooler
Add-ons
Hemofilter
CRRT/Prisma
7

## Slide 8
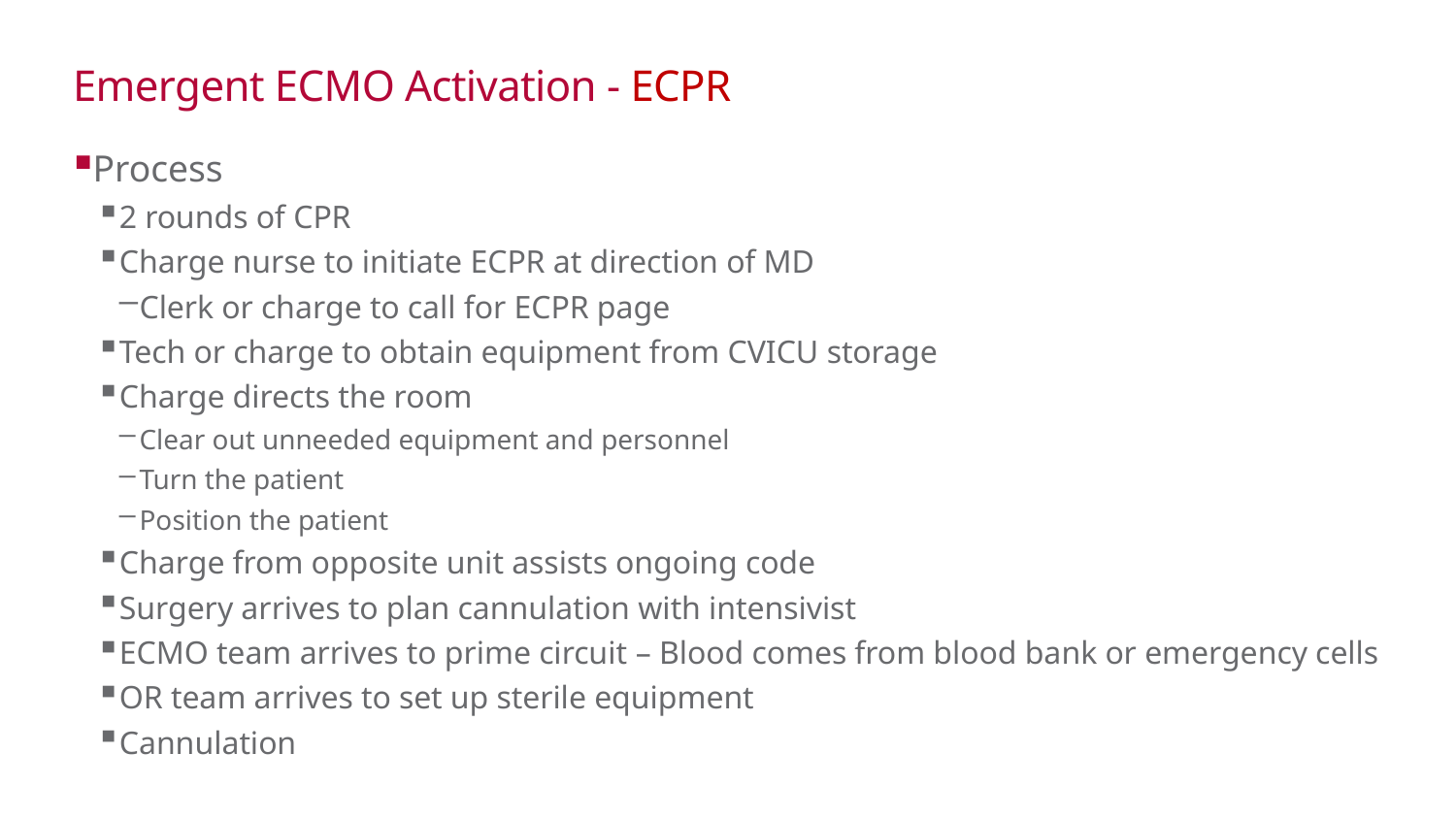

# Emergent ECMO Activation - ECPR
Process
2 rounds of CPR
Charge nurse to initiate ECPR at direction of MD
Clerk or charge to call for ECPR page
Tech or charge to obtain equipment from CVICU storage
Charge directs the room
Clear out unneeded equipment and personnel
Turn the patient
Position the patient
Charge from opposite unit assists ongoing code
Surgery arrives to plan cannulation with intensivist
ECMO team arrives to prime circuit – Blood comes from blood bank or emergency cells
OR team arrives to set up sterile equipment
Cannulation

## Slide 9
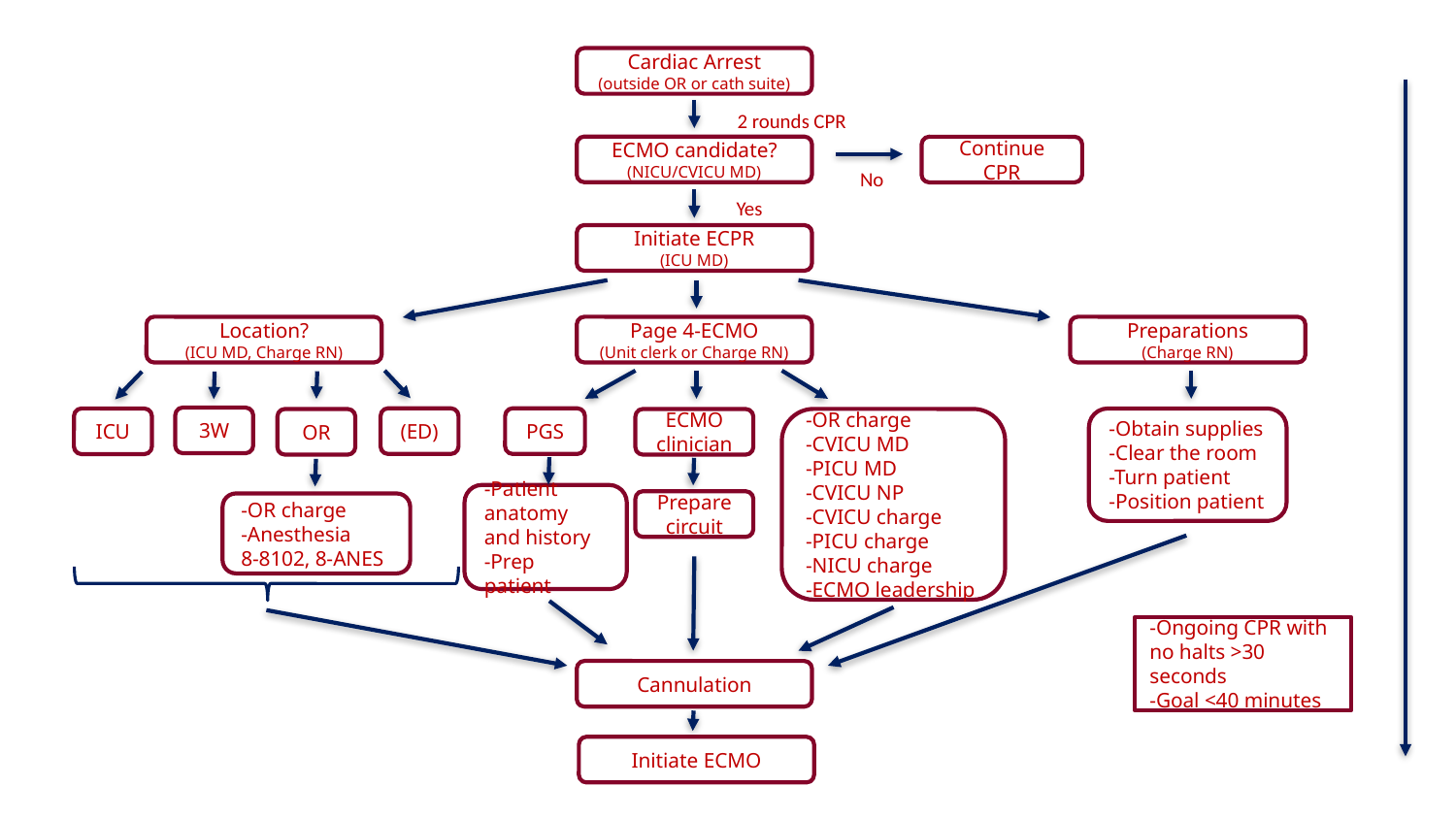

Cardiac Arrest
(outside OR or cath suite)
2 rounds CPR
ECMO candidate?
(NICU/CVICU MD)
Continue CPR
No
Yes
Initiate ECPR
(ICU MD)
Location?
(ICU MD, Charge RN)
Page 4-ECMO
(Unit clerk or Charge RN)
Preparations
(Charge RN)
3W
(ED)
PGS
ICU
-Obtain supplies
-Clear the room
-Turn patient
-Position patient
OR
ECMO
clinician
-OR charge
-CVICU MD
-PICU MD
-CVICU NP
-CVICU charge
-PICU charge
-NICU charge
-ECMO leadership
-Patient anatomy and history
-Prep patient
Prepare circuit
-OR charge
-Anesthesia
8-8102, 8-ANES
-Ongoing CPR with no halts >30 seconds
-Goal <40 minutes
Cannulation
Initiate ECMO

## Slide 10
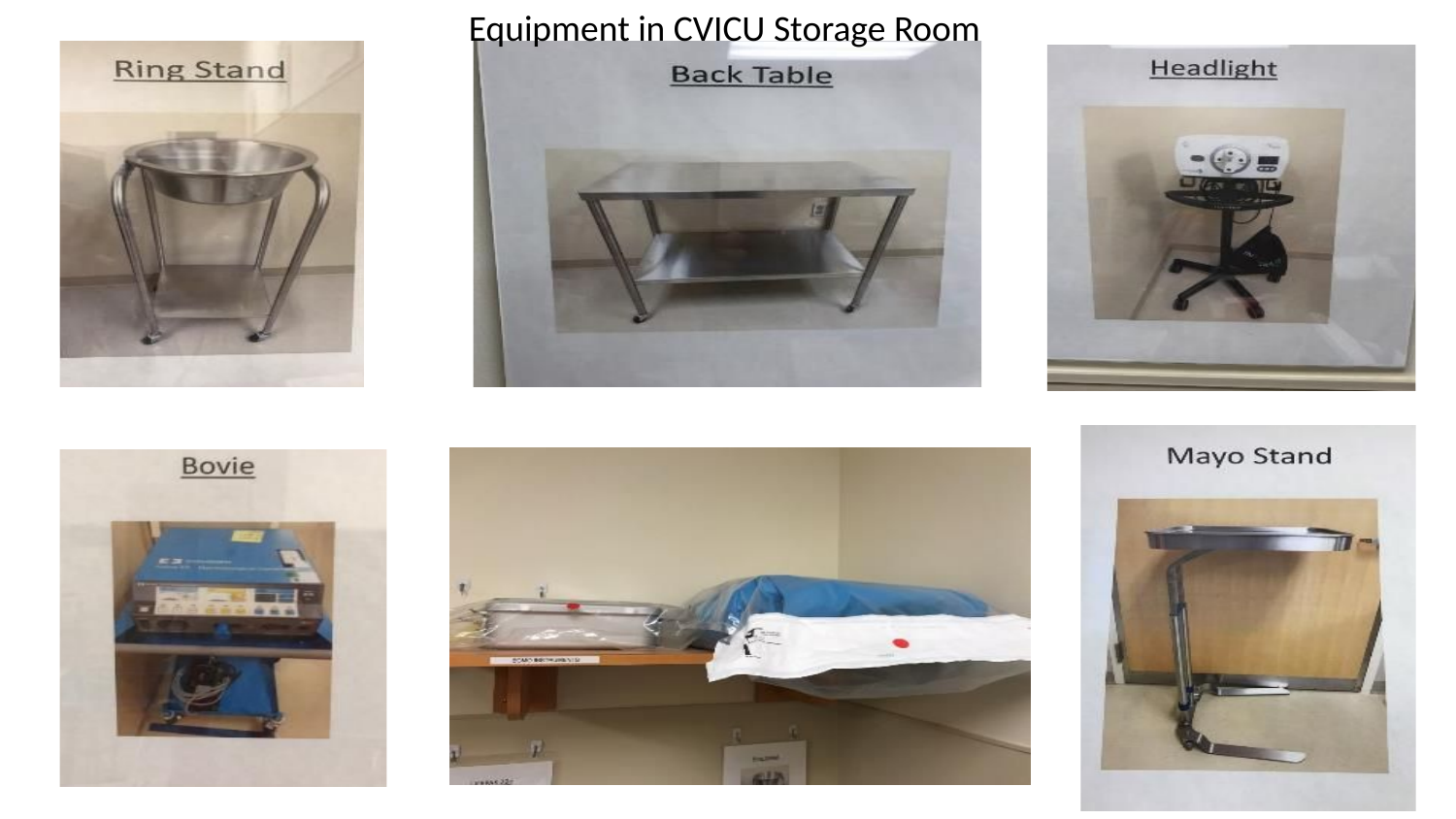

Equipment in CVICU Storage Room

## Slide 11
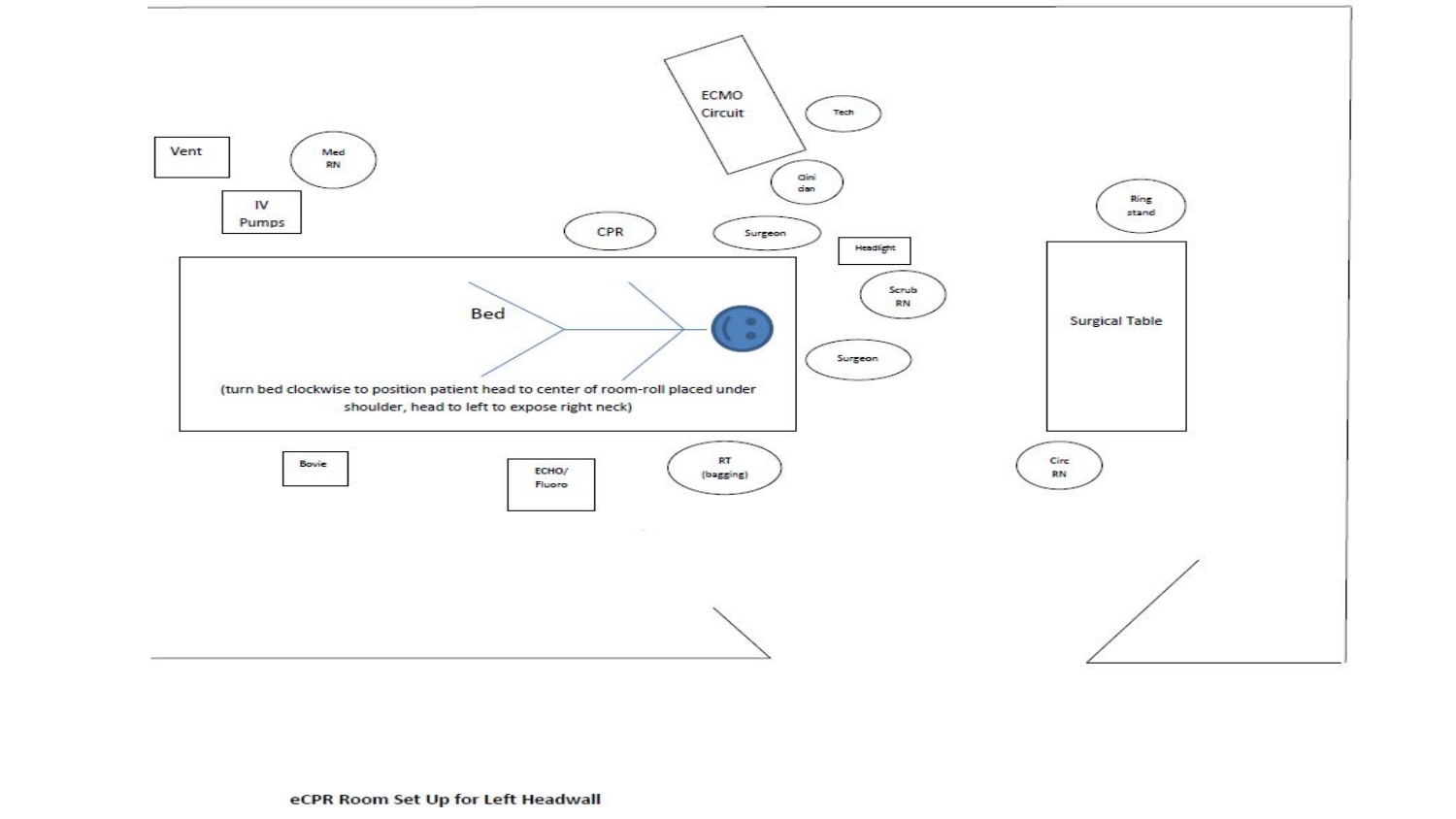

## Slide 12
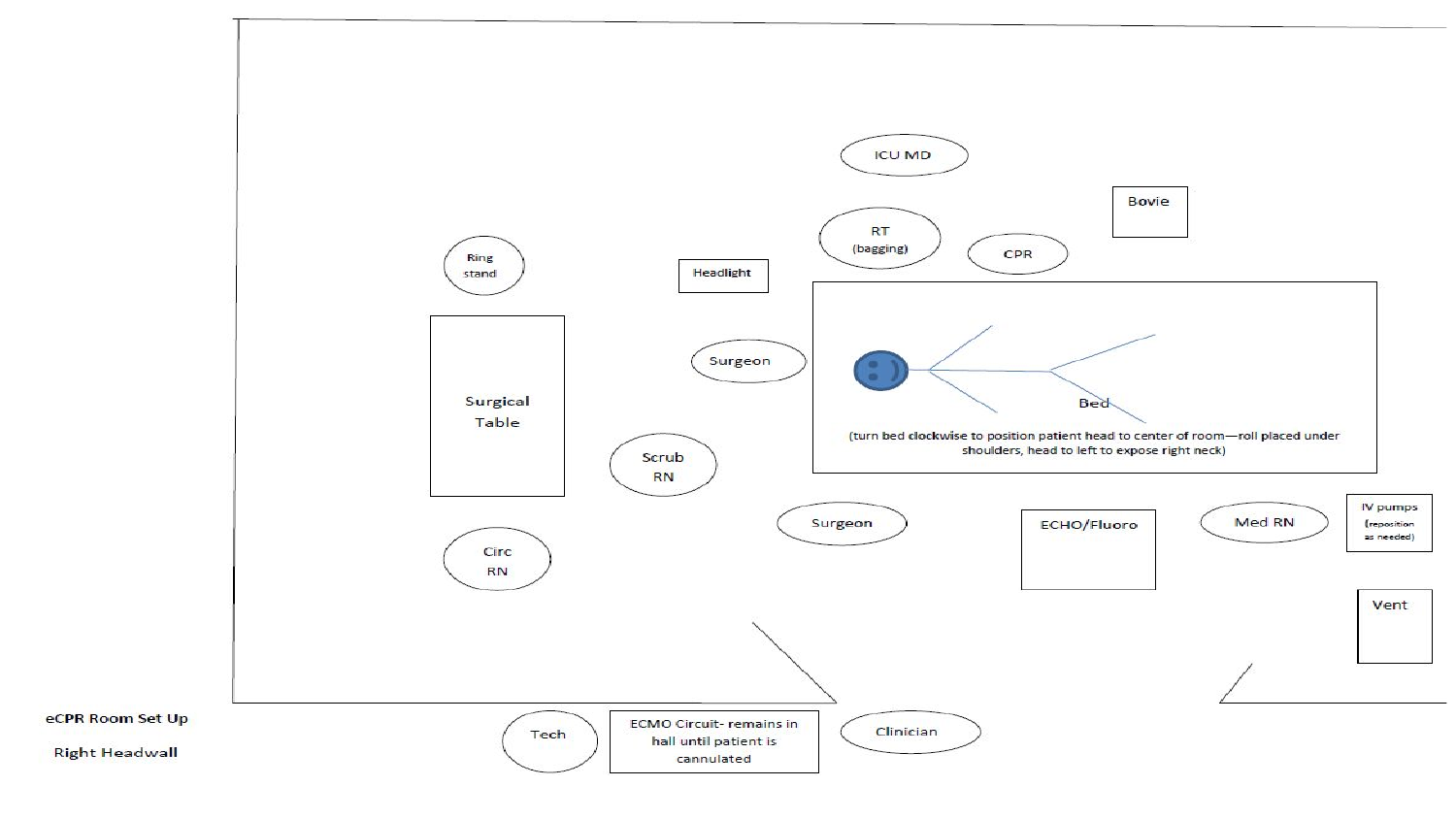

## Slide 13
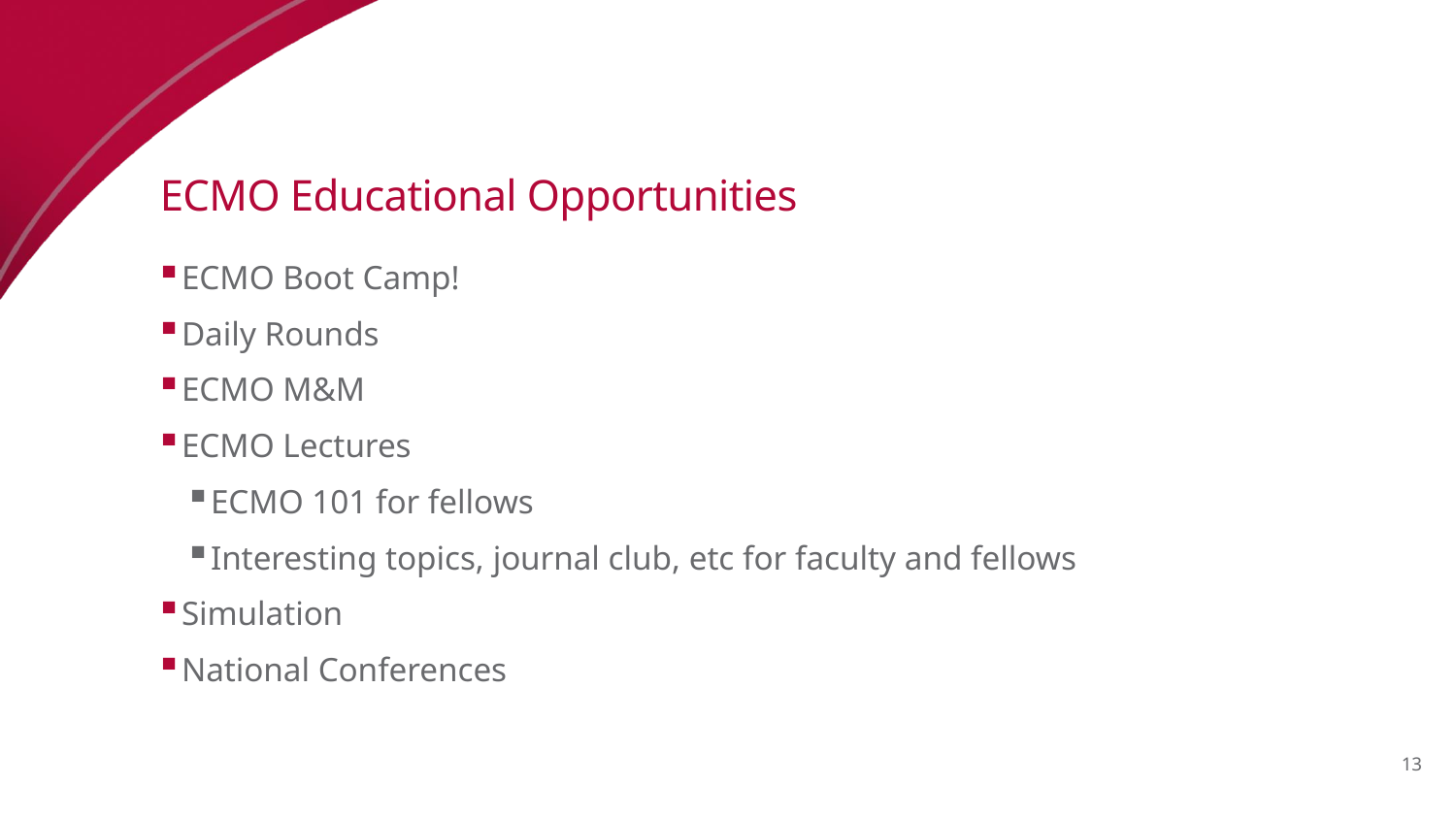

# ECMO Educational Opportunities
ECMO Boot Camp!
Daily Rounds
ECMO M&M
ECMO Lectures
ECMO 101 for fellows
Interesting topics, journal club, etc for faculty and fellows
Simulation
National Conferences
13
