# Supplementary material for: A Multimodal Educational Boot Camp for Training Fellows in Pediatric Extracorporeal Membrane Oxygenation (ECMO)
Source: MedEdPORTAL. 2024 Oct 17;20:11455. doi: 10.15766/mep_2374-8265.11455 (PMC11485016; doi:10.15766/mep_2374-8265.11455)
Supplement: Supplementary file 1 — Pneumothorax Simulation Case.docxECMO Pump Failure Simulation Case.docxCircuit Pressures Chart.docxTabletop ECMO Puzzle.pdfSample Agenda.docxIntroduction to ECMO.pptxECMO Knowledge Quiz.docxCircuit Components - Blank.pdfCircuit Components - Answers.docxCircuit Pressures Chart - Answers.docxPostsurvey.docx [file mep_2374-8265.11455-s001.zip › G. ECMO Knowledge Quiz.docx]

**Pediatric ECMO Boot Camp Test**

1. An increase in pre-membrane and post-membrane pressure is indicative of:
   1. Thrombosis or kinking of the venous side of the circuit
   2. Thrombosis or kinking of the arterial side of the circuit
   3. A severe pump malfunction, requiring the pump to be changed out
   4. An elevated platelet count
2. The transmembrane pressure is determined by:
   1. Inlet – outlet pressure
   2. Inlet + outlet pressure
   3. Outlet – inlet pressure
   4. Outlet/Inlet pressure
3. The membrane oxygenator sweep gas functions most like which ventilator parameter?
   1. Rate
   2. PIP
   3. PEEP
   4. FiO_2_
4. Which of the following typically has the greatest impact on the oxygen delivery in VA ECMO?
   1. ECMO circuit FiO_2_
   2. Post-oxygenator PaO_2_
   3. Oxygen uptake through the native lung
   4. ECMO circuit flow
5. The best indicator or measurement to determine adequate global oxygen delivery in the ECMO patient is:
   1. Post membrane oxygenator pO2
   2. Arterial oxygen saturation
   3. Arterial blood gas from the patient
   4. Mixed venous oxygen saturation
6. What blood pressure description is most commonly seen during the initiation of ECMO support?
   1. Hypertension
   2. Hypotension
   3. Normotension
   4. None of the above
7. What order are the pressure monitors located, beginning at the venous access cannula:
   1. 1, 2, 3, 4
   2. 4, 3, 2, 1
   3. 1, 3, 2, 4
   4. 2, 1, 3, 4
8. Identify the following piece of ECMO circuitry:
   1. Heat exchanger
   2. Membrane oxygenator
   3. Hemofilter
   4. Bladder/reservoir


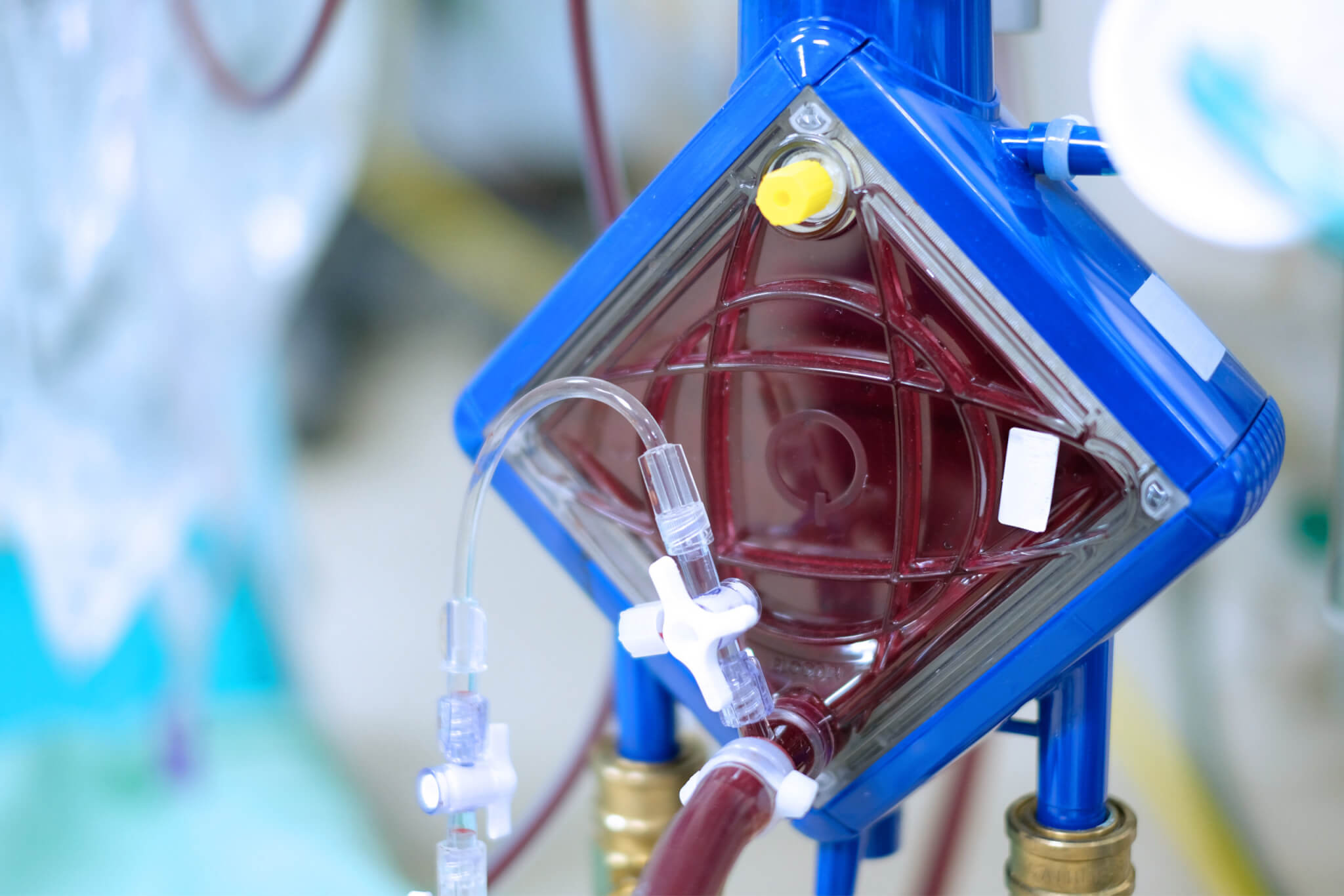


Answer Key:

1. b

2. a

3. a

4. d

5. d

6. b

7. c

8. b
