# Supplementary material for: A Multimodal Educational Boot Camp for Training Fellows in Pediatric Extracorporeal Membrane Oxygenation (ECMO)
Source: MedEdPORTAL. 2024 Oct 17;20:11455. doi: 10.15766/mep_2374-8265.11455 (PMC11485016; doi:10.15766/mep_2374-8265.11455)
Supplement: Supplementary file 1 — Pneumothorax Simulation Case.docxECMO Pump Failure Simulation Case.docxCircuit Pressures Chart.docxTabletop ECMO Puzzle.pdfSample Agenda.docxIntroduction to ECMO.pptxECMO Knowledge Quiz.docxCircuit Components - Blank.pdfCircuit Components - Answers.docxCircuit Pressures Chart - Answers.docxPostsurvey.docx [file mep_2374-8265.11455-s001.zip › I. Circuit Components - Answers.docx]

ECMO Circuit Components – ANSWERS

1. bridge (connection between venous and arterial side of circuit, can be used for troubleshooting)
2. arterial cannula
3. venous cannula
4. RPM/flow control knob
5. blood flow through circuit
6. RPM
7. sweep flow (controls O2 capacity of oxygenator)
8. blender (controls FiO2)
9. venous pressure (P1, measured prior to pump, most important for overall circuit)
10. pre-oxygenator pressure (P2)
11. post-oxygenator pressure (P4)
12. bladder pressure (P3, buffer for negative pressure, see #17)
13. heat exchanger (maintains patient normothermia)
14. centrifugal pump
15. sweep gas (green tubing that feeds into the oxygenator)
16. oxygenator
17. bladder (venous compliance chamber, may not be present on larger circuits)
18. bubble detector on arterial arm
19. pressure transducers
