# Supplementary material for: A Multimodal Educational Boot Camp for Training Fellows in Pediatric Extracorporeal Membrane Oxygenation (ECMO)
Source: MedEdPORTAL. 2024 Oct 17;20:11455. doi: 10.15766/mep_2374-8265.11455 (PMC11485016; doi:10.15766/mep_2374-8265.11455)
Supplement: Supplementary file 1 — Pneumothorax Simulation Case.docxECMO Pump Failure Simulation Case.docxCircuit Pressures Chart.docxTabletop ECMO Puzzle.pdfSample Agenda.docxIntroduction to ECMO.pptxECMO Knowledge Quiz.docxCircuit Components - Blank.pdfCircuit Components - Answers.docxCircuit Pressures Chart - Answers.docxPostsurvey.docx [file mep_2374-8265.11455-s001.zip › K. Postsurvey.docx]

**Post Boot Camp Evaluation**

Please provide your responses relative to the statements provided below, with 1 indicating **Strongly Disagree** with the statement and 5 indicating **Strongly Agree**

|  | Strongly Disagree | Disagree | Undecided | Agree | Strongly Agree |
| --- | --- | --- | --- | --- | --- |
|  | 1 | 2 | 3 | 4 | 5 |
| 1. The group lecture with hands-on circuit demonstration was beneficial in becoming familiarized with the circuit components |  |  |  |  |  |
| 2. The circuit components worksheet was a helpful tool to understand how the circuit works |  |  |  |  |  |
| 3. The ECMO circuit pressure chart/group exercise was helpful in enhancing understanding ECMO circuit pressure physiology |  |  |  |  |  |
| 4. The tabletop circuit exercise was useful for enhancing skills in ECMO circuit construction |  |  |  |  |  |
| 5. The high-fidelity simulation scenario (pump failure) improved my self-confidence and comfort level with ECMO management |  |  |  |  |  |
| 6. The high-fidelity simulation scenario (pneumothorax) improved my self-confidence and comfort level with ECMO management |  |  |  |  |  |
| 7. The boot camp was overall helpful in improving my confidence and comfort in understanding and troubleshooting ECMO emergencies |  |  |  |  |  |

**Free Response Questions**

1. What was most useful part about the boot camp?
2. What was the least useful part about the boot camp?
3. How could this boot camp be improved?
4. Was the inclusion of PICU/CVICU fellows, NICU fellows, cardiology fellows, and pediatric surgery/cardiac surgery fellows in the boot camp beneficial? If so, how? If not, why not?
5. Did the faculty-led sessions facilitate an effective and engaging learning environment?

Other comments?
