# Supplementary material for: A Multimodal Educational Boot Camp for Training Fellows in Pediatric Extracorporeal Membrane Oxygenation (ECMO)
Source: MedEdPORTAL. 2024 Oct 17;20:11455. doi: 10.15766/mep_2374-8265.11455 (PMC11485016; doi:10.15766/mep_2374-8265.11455)
Supplement: Supplementary file 1 — Pneumothorax Simulation Case.docxECMO Pump Failure Simulation Case.docxCircuit Pressures Chart.docxTabletop ECMO Puzzle.pdfSample Agenda.docxIntroduction to ECMO.pptxECMO Knowledge Quiz.docxCircuit Components - Blank.pdfCircuit Components - Answers.docxCircuit Pressures Chart - Answers.docxPostsurvey.docx [file mep_2374-8265.11455-s001.zip › _Educational Summary Report_11455.pdf]

MedEdPORTAL® The AAMC Journal of Teaching and Learning Resources

Original Publication Open Access

A Multimodal Educational Boot Camp for Training Fellows in Pediatric Extracorporeal Membrane Oxygenation (ECMO)

Yujin Park, MD, MS*, Gail Hocutt, MSN, RNC-NIC, Elizabeth Wetzel, MD, Nathan Swinger, MD, Kellie Pearson, RRT, NPS, Kamal Abulebda, MD, Brian Gray, MD

*Corresponding author: yp14@iu.edu

Abstract

Introduction: Pediatric extracorporeal membrane oxygenation (ECMO) management presents unique challenges in acute care settings, requiring specialized expertise to manage critically ill children. Medical and surgical fellows often manage these patients, but prior residency training rarely provides sufficient ECMO exposure. We developed and evaluated a multimodal pediatric ECMO boot camp for new fellows. **Methods:** This boot camp was implemented during 5-hour sessions in August 2021, August 2022, and August 2023. The curriculum included a 45-minute introductory didactics session, 30-minute hands-on circuit demonstration, and four 30-minute small-group activity stations. To assess knowledge acquisition, pre- and posttests were administered; participants also completed a post–boot camp survey to evaluate their confidence and provide feedback. **Results:** Forty-nine participants completed the boot camp, including 18 critical care, four cardiology, 11 pediatric surgery, 12 cardiothoracic surgery, and four pediatric emergency medicine fellows. Pre- and posttests demonstrated significant improvement in knowledge of ECMO circuit components and pressures (56% vs. 76%, $p < .001$). All of our participants agreed or strongly agreed that participating in the boot camp increased their confidence in troubleshooting ECMO emergencies. The inclusion of fellows from various clinical disciplines, offering a rich diversity of perspectives, was particularly valued by participants. **Discussion:** Our results demonstrate the feasibility and effectiveness of establishing a pediatric ECMO boot camp to train new surgical and medical fellows. The curriculum not only improved ECMO knowledge but also boosted learners' confidence in managing ECMO-related challenges.

Keywords
Boot Camp, ECMO, Fellowship, Clinical/Procedural Skills Training, Pediatric Critical Care Medicine, Simulation, Surgery - Pediatric

Educational Objectives

By the end of this activity, learners will be able to:

1. Name the various components of the extracorporeal membrane oxygenation (ECMO) circuit, including the oxygenator, arterial cannula, venous cannula, pump, tubing, and pressure monitoring devices, and identify each component's function.
2. Describe the anticipated changes (increase vs. decrease) in ECMO circuit pressures that occur in response to different clinical scenarios.
3. Evaluate a patient on ECMO in a simulation scenario and use data from circuit pressures and other clinical information to diagnose and manage ECMO-related emergencies.
4. Report confidence in the management of ECMO-related emergencies.

Citation:
Park Y, Hocutt G, Wetzel E, et al. A multimodal educational boot camp for training fellows in pediatric extracorporeal membrane oxygenation (ECMO). *MedEdPORTAL.* 2024;20:11455.
https://doi.org/10.15766/mep_2374-8265.11455

Introduction

Pediatric extracorporeal membrane oxygenation (ECMO) is a therapy that provides cardiac and pulmonary support in the setting of heart or lung failure. This treatment involves cannulating a patient's central blood vessels to facilitate external blood circulation and oxygenation before returning blood to the body. Within the landscape of critical care, medical and surgical fellows play indispensable roles in managing acutely ill ECMO patients. However, conventional residency training rarely imparts comprehensive ECMO knowledge, and fellowship programs also vary in ECMO exposure, highlighting a significant learning gap

Copyright © 2024 Park et al. This is an open-access publication distributed under the terms of the Creative Commons Attribution license.

that needs to be addressed.[1] We sought to address this gap by implementing and assessing a multimodal pediatric ECMO boot camp. Our target audience was first-year fellows at the start of their respective fellowships.

Despite the increasing utilization of ECMO therapy over recent decades, a lack of standardized ECMO training persists.[2,3] The Extracorporeal Life Support Organization has published guidelines for training ECMO specialists—individuals adept at managing both the ECMO system and the clinical demands of ECMO patients under the guidance of licensed ECMO-trained physicians[4]—but significant variation exists in how these practices have been adopted at various ECMO centers.[5] A recent survey of adult critical care fellowship program directors unveiled the need for enhanced educational methods, as many programs lack dedicated training initiatives for ECMO knowledge.[1]

This boot camp was designed to provide an opportunity for fellows to have their first ECMO immersion and work collaboratively with other subspecialty fellows in a simulated environment. We utilized a multimodal, innovative approach to pediatric ECMO training for fellows combining multiple educational methods (high-fidelity simulation, guided circuit pressures activity, tabletop puzzle, and didactics) to improve baseline knowledge of managing and troubleshooting ECMO emergencies in an engaging and effective manner.

Medical simulation has proven its efficacy in diverse realms of medical education, encompassing interprofessional team training and error management and augmenting health care providers' skills, knowledge, and behaviors.[6-9] Central to our boot camp is the application of high-fidelity simulation as an evaluative and instructional tool. Previous studies have underscored simulation-based training's superiority over traditional methods for novice critical care fellows, reinforcing our decision to incorporate high-fidelity simulation scenarios into our boot camp. This approach also nurtures multidisciplinary teamwork, a cornerstone of effective pediatric ECMO patient management.[10-13]

A defining facet of our boot camp is its inclusivity, drawing fellows from various disciplines—surgery, critical care, medical specialties, and emergency medicine—to emphasize the significance of multiprofessional collaboration. To our knowledge, our work stands as a unique contribution to *MedEdPORTAL*, with no prior publications documenting similar endeavors. Furthermore, the topics we selected for our boot camp, including comprehension of circuit components, interpretation of circuit pressures within the framework of ECMO physiology, and ECMO emergency management, have been identified by ECMO experts as pivotal content for critical care fellows.[3] Our hope is that our boot camp can accelerate clinical proficiency of new fellows in ECMO, serving as a catalyst for improved cognitive, technical, and behavioral competencies.

Methods

Development

We designed a comprehensive 5-hour multimodal educational boot camp in pediatric ECMO for junior clinical fellows across various disciplines. The educational content of the boot camp was crafted by experts in ECMO and simulation-based education. To ensure facilitators had appropriate content knowledge and could successfully execute each boot camp component, facilitators were either physicians with extensive ECMO expertise or ECMO clinical specialists (nurses and respiratory therapists with advanced ECMO training). Notably, no prerequisite ECMO-related knowledge was needed or assumed for the participating learners.

The boot camp included a rotation of small groups through four distinct stations. These stations featured a guided discussion addressing ECMO circuit pressures via a worksheet (used with permission from Mark Todd, the Program Manager at SickKids Toronto), two high-fidelity simulation scenarios developed internally at our institution by a collaborative effort of ECMO and simulation content experts, and a tabletop puzzle involving circuit components that was initially adapted from an ECMO simulation workshop presented at the 2019 International Pediatric Simulation Symposia and Workshops conference.

Equipment/Environment

For the optimal realization of this boot camp, we leveraged resources and spaces at Riley Hospital for Children in Indianapolis, Indiana. High-fidelity mannequins (SimJunior and SimNewB) by Laerdal, alongside two ECMO circuits, were instrumental in conducting the two simulation sessions. To ensure the fidelity of the simulations, we employed actual patient rooms to recreate authentic clinical settings. Supplementary conference rooms were utilized for the remaining boot camp stations. Each simulation was facilitated by ECMO content experts proficient in postsimulation debriefing. Simulation technicians preprogrammed all simulations, providing vital signs, lab reports, and imaging upon learners' request. The simulations commenced with participants receiving a brief patient history before entering the simulation room. An ECMO specialist or expert was embedded within each scenario, serving as a bedside ECMO nurse. This embedded nurse controlled patient vital signs and ECMO circuit pressures, making changes in response to

Copyright © 2024 Park et al. This is an open-access publication distributed under the terms of the Creative Commons Attribution license.

participants' actions. Further details for the equipment and setup of both simulation scenarios are included in Appendices A and B.

For the guided circuit pressures worksheet activity, a small classroom space was requisite, with each participant equipped with a printed copy of the blank chart (Appendix C). The tabletop ECMO circuit component puzzle necessitated the printing, cutting, and lamination of individual puzzle pieces (Appendix D). An ample table was provided to accommodate participants as they reconstructed the puzzle into a complete ECMO circuit.

Personnel

The personnel required to implement the boot camp included four facilitators and a boot camp coordinator. Our facilitators consisted of a pediatric surgeon with ECMO fellowship training, who guided the circuit pressure worksheet; two critical care physicians experienced in managing ECMO patients, who conducted simulations and tabletop circuit exercises; and the ECMO program nurse manager, who also participated in the simulations. The coordinator was in charge of coordinating all activities and ensuring smooth execution of the boot camp.

Implementation

We implemented our pediatric ECMO boot camp as a stand-alone educational activity during the first month of fellowship training. The boot camp was structured as described in the agenda (Appendix E), starting with a brief 15-minute program overview (Appendix F) followed by 15 minutes to complete the pre–boot camp ECMO knowledge assessment (Appendix G). Please note that Appendix F may need to be further adapted prior to use based on local team structures and practices. A portion of the large-group session consisted of a 30-minute live demonstration and identification activity of ECMO circuit components. We provided participants with a worksheet (Appendices H and I) consisting of a photograph of an ECMO circuit with blank spaces to fill in the names of each component as it was reviewed during the session.

At the conclusion of the circuit component identification activity, we divided the participants into four smaller groups, each consisting of four or five fellows from a mix of different specialties. We intentionally formed the groups in this way to encourage collaboration between the various clinical disciplines represented.

The small groups then rotated through four activity stations, with 30 minutes allotted to each station and an appropriate amount of time given to transition between stations. The activity stations included a guided ECMO circuit pressures worksheet, two high fidelity simulation scenarios, and a tabletop puzzle of circuit components, described below.

Guided ECMO circuit pressures worksheet (Appendices C and J): At this station, we provided the fellows with a blank chart (Appendix C) with important circuit pressures along the top row and various clinical scenarios relevant to ECMO listed down the left-hand column. One of our ECMO experts led the small group in a discussion going row by row and describing how each clinical scenario might be expected to alter the ECMO circuit pressures. The completed chart is provided in Appendix J. This activity was designed to help fellows recognize patterns in circuit pressures that might be seen in different ECMO-related clinical problems and reinforce their understanding of the underlying physiology to assist them with troubleshooting ECMO emergencies.

Simulation stations (Appendices A and B): We had two separate high-fidelity simulation stations with scenarios for ECMO troubleshooting developed by our local ECMO experts. Each station was facilitated by either a faculty clinical ECMO expert or an ECMO specialist embedded in the case as a bedside ECMO nurse, with access to control the vital signs and ECMO pressures displayed on the monitor. The facilitator was familiarized with the simulation script prior to the day of the boot camp. After entering the room as a team, the participants were presented with a brief patient history and collaborated to identify the clinical problem and decide on an appropriate management solution. At the conclusion of the case, the facilitator led a focused debrief discussion.

Tabletop puzzle of circuit components (Appendix D): Preparation for this activity involved printing and cutting out the circuit component images provided in Appendix D. We then laminated the paper puzzle pieces to allow for repeated use. This activity was overseen by a single facilitator and required a broad, flat surface on which to lay out the puzzle pieces. The puzzle was designed to be completed by a small group of two or three learners, so we provided two full sets of puzzle pieces and further divided each small group into two groups for this activity. The facilitator provided hints and feedback and directed the learners to consider the function of each component to help find its place in the circuit.

Debriefing

When all participants had rotated through all four activity stations, the group reconvened in a final, 30-minute, large-group session for a debriefing of the entire boot camp led by ECMO experts.

Copyright © 2024 Park et al. This is an open-access publication distributed under the terms of the Creative Commons Attribution license.

The participants were also asked to complete the post–boot camp knowledge quiz (Appendix G) and post–boot camp experience survey (Appendix K).

Assessment

We assessed the effectiveness of our pediatric ECMO boot camp both quantitatively and qualitatively, using a pre- and postcurriculum, cognitive, multiple-choice quiz developed by a team of ECMO experts at our institution (Appendix G) and a postcurriculum experience survey (Appendix K) regarding the usefulness of each boot camp component and self-rated confidence using a 5-point Likert scale (1 = *strongly disagree*, 5 = *strongly agree*). We additionally collected free-response qualitative written feedback. The critical action checklists for the simulation activities were developed through collaborative efforts of our institutional ECMO experts.

Results

We executed the boot camp three times over 3 years, with a total of 49 fellow participants, comprising 18 individuals specializing in critical care (eight pediatric, nine neonatal, and one cardiovascular), four in cardiology, 11 in pediatric surgery, 12 in cardiothoracic surgery, and four in pediatric emergency medicine. The results of the knowledge assessment suggested a significant improvement in ECMO knowledge, with the average pre–boot camp score of 56% increasing to an average post–boot camp score of 76% ($p < .001$). All participants completed the pre- and postassessments. One hundred percent of our participants agreed or strongly agreed (86% strongly agreed) that participating in the boot camp increased their confidence in troubleshooting ECMO emergencies. Additionally, the majority of participants either agreed or strongly agreed that each individual boot camp component proved valuable, with particular emphasis on the simulation scenarios, which were identified as the most beneficial (introduction with live demonstration of ECMO circuit: 100% agreed or strongly agreed, 75% strongly agreed; guided circuit pressures worksheet: 98% agreed or strongly agreed, 85% strongly agreed; tabletop puzzle: 98% agreed or strongly agreed, 69% strongly agreed; simulation scenarios: 100% agreed or strongly agreed, 69% strongly agreed). We collected qualitative feedback in the form of free responses, revealing that participants particularly commended the integration of fellows from diverse clinical disciplines. Fellows appreciated that each participant contributed “different insights during the simulation scenarios” and felt that the boot camp was “helpful in developing multidisciplinary connections” early in the year.

Discussion

Reflecting on the entire journey of developing, implementing, and evaluating our pediatric ECMO boot camp, it became evident to us that this endeavor was instrumental in establishing a process to start addressing a significant educational gap. The initial trigger behind the boot camp was the realization that despite the growing prominence of ECMO therapy, traditional residency training inadequately equipped junior clinical fellows with the necessary knowledge and confidence to manage ECMO emergencies. Experts in ECMO and simulation-based education contributed to the design of our comprehensive curriculum. The varied modalities, including high-fidelity simulation, guided circuit pressure activities, tabletop puzzles, and didactics, were strategically combined to create an engaging and meaningful learning experience.

Our project predominantly focused on levels 1 and 2 of the Kirkpatrick model[14] for evaluating educational programs, demonstrating that our participants not only derived satisfaction but also demonstrated measurable advancement in their ECMO knowledge foundation. Fellows reported an increase in their confidence with troubleshooting ECMO-related emergencies attributed to the multidisciplinary collaborative nature of the boot camp activities. Furthermore, a diverse multidisciplinary cohort of 49 participants spanning various clinical disciplines engaged in the boot camp, contributing to a rich learning environment.

Our results demonstrate that our boot camp is a promising start to bridging some knowledge gaps in pediatric ECMO among junior clinical fellows. By offering a unique blend of didactic and hands-on experiences, we empowered participants to become immersed in ECMO-related emergencies with improved self-perceived competency and confidence. Furthermore, the positive comments from participants regarding the multidisciplinary collaborative nature of the boot camp support its efficacy in enhancing teamwork and fostering interdisciplinary understanding, which are crucial aspects of managing complex cases like pediatric ECMO.

The evaluation of our boot camp does have some limitations. The results were drawn from a relatively small sample size and conducted within a specific hospital setting. This could limit the generalizability of our findings to other institutions with different demographics, resources, and educational structures. Although most of our boot camp activities can be recreated using the provided appendices, we recognize that our simulation scenarios include specific ECMO devices such as an ECMO simulator that some centers may not have access to. To address this

Copyright © 2024 Park et al. This is an open-access publication distributed under the terms of the Creative Commons Attribution license.

issue, for each simulation scenario we provide flow sheets with circuit pressure values that learners can be given based on their actions to allow them to troubleshoot even in the absence of an advanced ECMO simulator. Our simulation scenarios ideally involve either an actual ECMO technician or a bedside nurse as the embedded participant, but we have also had success with the instructor filling this role when additional ECMO team members were unavailable to participate.

Furthermore, while we aimed to evaluate the effectiveness of the boot camp within the framework of the Kirkpatrick model, our evaluation primarily focused on participants' perceptions and knowledge improvement, without assessing behavior change or patient outcomes. Future work will provide more insight into higher-level learning outcomes. We did not explore the long-term retention of knowledge improvement in this boot camp since all learning outcomes were collected immediately after the boot camp's completion. Another limitation of our project is that it was not designed to compare the effectiveness of our boot camp against more traditional lecture or textbook-based teaching.

The success of the multidisciplinary approach within fellowship programs in our boot camp could serve as a model for integrating collaborative learning into various medical education programs. Additionally, the incorporation of blended educational modalities within a short span underscores the feasibility of such approaches in other domains of medical education. Scaling this boot camp to other institutions nationally or internationally opens avenues for wider dissemination of ECMO education. This work can serve as a supplementary asset alongside the efforts undertaken by other institutions and agencies to advance the field of ECMO, facilitating the promotion of scientific understanding and the implementation of more comprehensive educational curricula. Another potential future direction for our boot camp would be an expansion to include interdisciplinary training by involving ECMO perfusionists and ECMO RNs in the simulation scenarios.

In summary, our project demonstrated the successful implementation of a boot camp designed to train junior clinical fellows and start to address knowledge gaps in pediatric ECMO. The favorable outcomes encompassing enhanced knowledge, improved confidence, and fortified multidisciplinary collaboration are strong evidence of the success of our strategy. While we acknowledge the constraints and avenues for further improvement, our efforts establish a solid foundation for future initiatives in ECMO education, carrying implications for the enhancement of curriculum design and the shaping of educational policy.

Appendices

A. Pneumothorax Simulation Case.docx
B. ECMO Pump Failure Simulation Case.docx
C. Circuit Pressures Chart.docx
D. Tabletop ECMO Puzzle.pdf
E. Sample Agenda.docx
F. Introduction to ECMO.pptx
G. ECMO Knowledge Quiz.docx
H. Circuit Components - Blank.pdf
I. Circuit Components - Answers.docx
J. Circuit Pressures Chart - Answers.docx
K. Postsurvey.docx

All appendices are peer reviewed as integral parts of the Original Publication.

Yujin Park, MD, MS: Sixth-Year General Surgery Resident, Department of Surgery, Indiana University School of Medicine; ORCID: https://orcid.org/0000-0001-6542-5761

Gail Hocutt, MSN, RNC-NIC: ECMO Program Director, ECMO Program Management, Division of Pediatric Surgery, Department of Surgery, Riley Hospital for Children at Indiana University Health

Elizabeth Wetzel, MD: Assistant Professor, Division of Neonatal-Perinatal Medicine, Department of Pediatrics, Indiana University School of Medicine and Riley Hospital for Children at Indiana University Health

Nathan Swinger, MD: Assistant Professor, Division of Pediatric Critical Care Medicine, Department of Pediatrics, Indiana University School of Medicine and Riley Hospital for Children at Indiana University Health

Kellie Pearson, RRT, NPS: Simulation Program Manager, Division of Pediatric Critical Care Medicine, Department of Pediatrics, Simulation Program, Indiana University School of Medicine and Riley Hospital for Children at Indiana University Health

Kamal Abulebda, MD: Associate Professor, Division of Pediatric Critical Care Medicine, Department of Pediatrics, Indiana University School of Medicine and Riley Hospital for Children at Indiana University Health

Brian Gray, MD: Assistant Professor and Co-director of ECMO Program, Division of Pediatric Surgery, Department of Surgery, Indiana University School of Medicine and Riley Hospital for Children at Indiana University Health

Disclosures
None to report.

Funding/Support
None to report.

Copyright © 2024 Park et al. This is an open-access publication distributed under the terms of the Creative Commons Attribution license.

Ethical Approval

Reported as not applicable.

References

1. Cook MR, Badulak J, Coruh B, et al. Fellowship training in extracorporeal life support: characterization and educational needs assessment. *J Crit Care*. 2018;46:159-161. https://doi.org/10.1016/j.jcrc.2018.03.023
2. Bartlett RH, Gazzaniga AB, Fong SW, Jefferies MR, Roohk HV, Haiduc N. Extracorporeal membrane oxygenator support for cardiopulmonary failure: experience in 28 cases. *J Thorac Cardiovasc Surg*. 1977;73(3):375-386. https://doi.org/10.1016/S0022-5223(19)39916-7
3. Crannell WC, Zakhary B, Hamilton H, Brasel K, Zonies D. Design of an entrustable professional activity for adult extracorporeal membrane oxygenation. *Surg Open Sci*. 2020;2(1):42-45. https://doi.org/10.1016/j.sopen.2019.09.001
4. *ELSO Guidelines for Training and Continuing Education of ECMO Specialists*. Extracorporeal Life Support Organization; 2010. Accessed September 24, 2024. https://www.elso.org/portals/0/igd/archive/filemanager/97000963d6cusersshyerdocumentselsoguidelinesfortrainingandcontinuingeducationofecmospecialists.pdf
5. Han PK, Purkey NJ, Kuo KW, et al. A scoping review and appraisal of extracorporeal membrane oxygenation education literature. *ATS Sch*. 2022;3(3):468-484. https://doi.org/10.34197/ats-scholar.2022-0058RE
6. Issenberg SB, Mcgaghie WC, Petrusa ER, Gordon DL, Scalese RJ. Features and uses of high-fidelity medical simulations that lead to effective learning: a BEME systematic review. *Med Teach*. 2005; 27(1):10-28. https://doi.org/10.1080/01421590500046924
7. McGaghie WC, Issenberg SB, Petrusa ER, Scalese RJ. A critical review of simulation-based medical education research: 2003–2009. *Med Educ*. 2010;44(1):50-63. https://doi.org/10.1111/j.1365-2923.2009.03547.x
8. Motola I, Devine LA, Chung HS, Sullivan JE, Issenberg SB. Simulation in healthcare education: a best evidence practical guide. AMEE Guide no. 82. *Med Teach*. 2013;35(10): e1511-e1530. https://doi.org/10.3109/0142159X.2013.818632
9. Lopreiato JO, Sawyer T. Simulation-based medical education in pediatrics. *Acad Pediatr*. 2015;15(2):134-142. https://doi.org/10.1016/j.acap.2014.10.010
10. Zakhary BM, Kam LM, Kaufman BS, Felner KJ. The utility of high-fidelity simulation for training critical care fellows in the management of extracorporeal membrane oxygenation emergencies: a randomized controlled trial. *Crit Care Med*. 2017;45(8):1367-1373. https://doi.org/10.1097/CCM.0000000000002437
11. Chan SY, Figueroa M, Spentzas T, Powell A, Holloway R, Shah S. Prospective assessment of novice learners in a simulation-based extracorporeal membrane oxygenation (ECMO) education program. *Pediatr Cardiol*. 2013;34(3):543-552. https://doi.org/10.1007/s00246-012-0490-6
12. Cheng A, Auerbach M, Hunt EA, et al. Designing and conducting simulation-based research. *Pediatrics*. 2014;133(6):1091-1101. https://doi.org/10.1542/peds.2013-3267
13. Khobrani A, Patel NH, George RL, McNinch NL, Ahmed RA. Pediatric trauma boot camp: a simulation curriculum and pilot study. *Emerg Med Int*. 2018;(1):7982315. https://doi.org/10.1155/2018/7982315
14. Craig RL, Bittel LR, eds. *Training and Development Handbook*. McGraw-Hill; 1967.

Received: December 15, 2023
Accepted: June 21, 2024
Published: October 17, 2024

Copyright © 2024 Park et al. This is an open-access publication distributed under the terms of the Creative Commons Attribution license.
