# Supplementary figures and images for: A Multimodal Educational Boot Camp for Training Fellows in Pediatric Extracorporeal Membrane Oxygenation (ECMO)
Source: MedEdPORTAL. 2024 Oct 17;20:11455. doi: 10.15766/mep_2374-8265.11455 (PMC11485016; doi:10.15766/mep_2374-8265.11455)

# ECMO Circuit Components

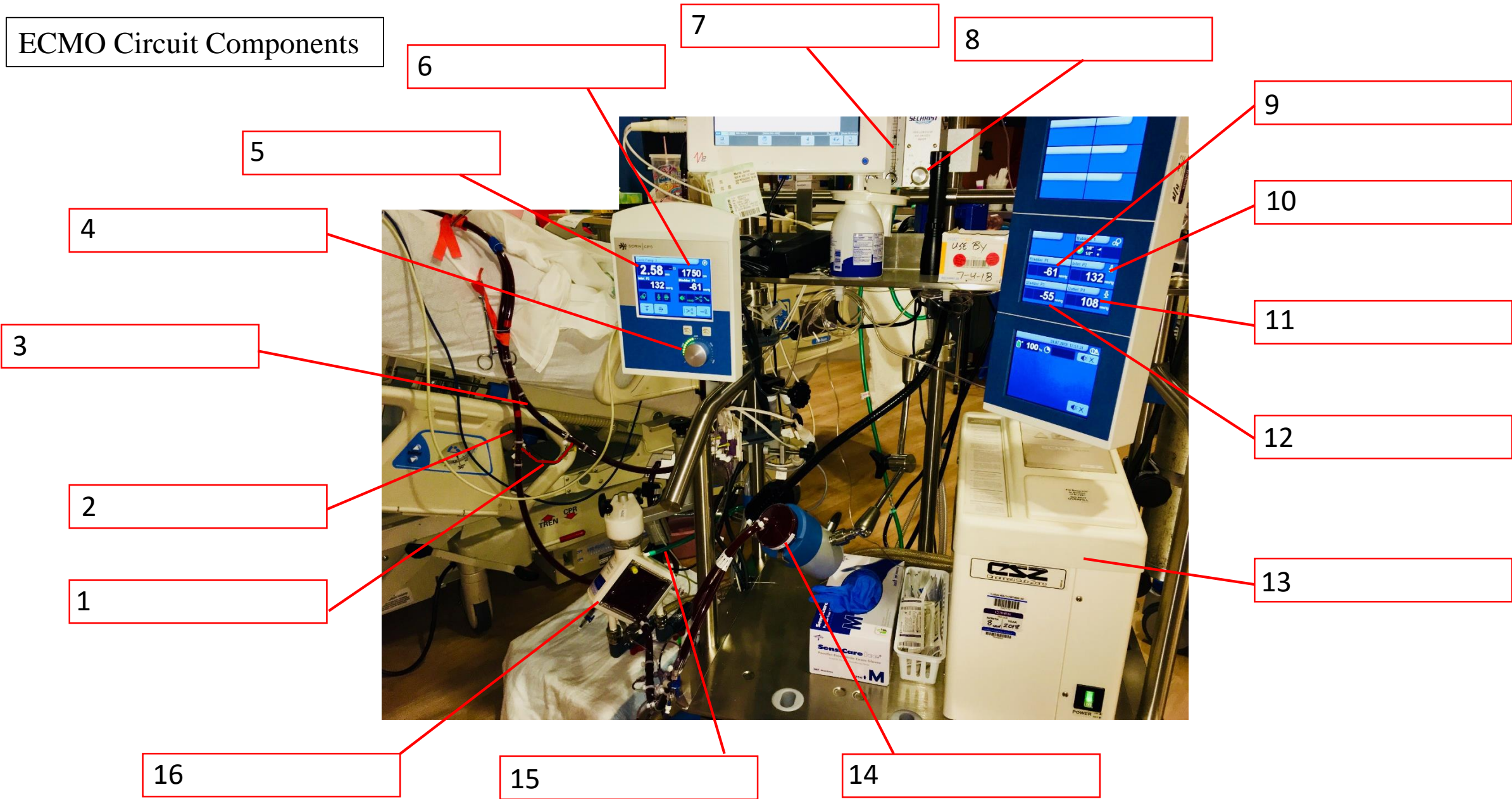

Recommended Activity Duration: 30 min

17

18

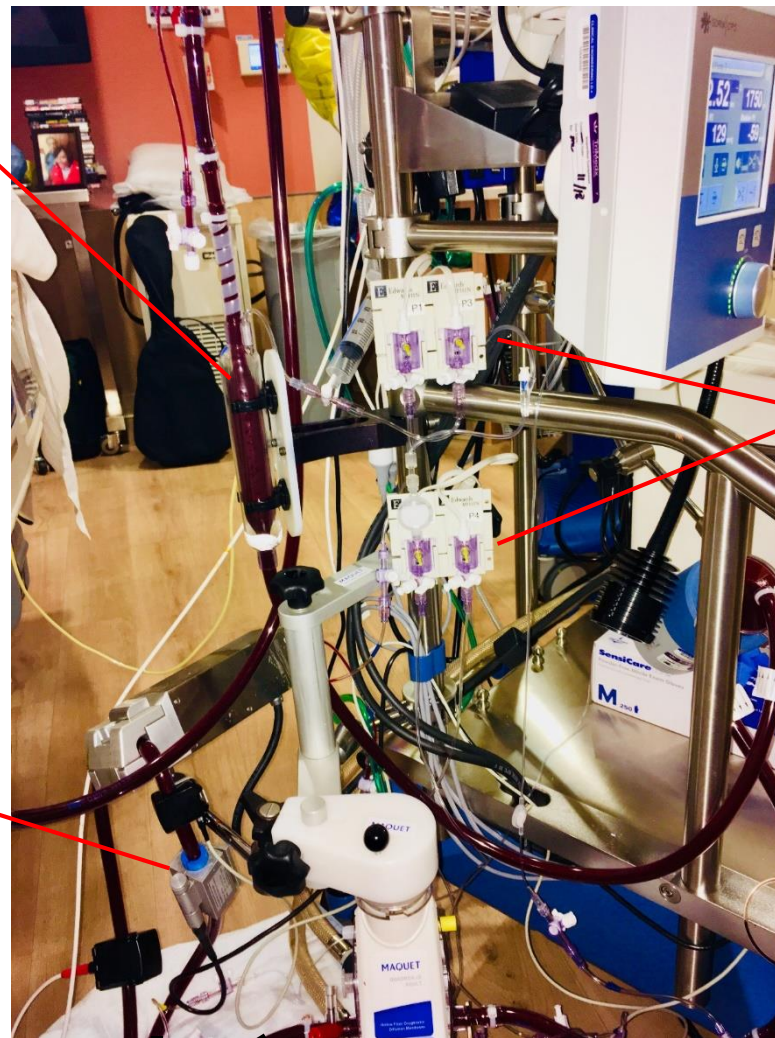

19

Supplement: Supplementary file 1 — Pneumothorax Simulation Case.docxECMO Pump Failure Simulation Case.docxCircuit Pressures Chart.docxTabletop ECMO Puzzle.pdfSample Agenda.docxIntroduction to ECMO.pptxECMO Knowledge Quiz.docxCircuit Components - Blank.pdfCircuit Components - Answers.docxCircuit Pressures Chart - Answers.docxPostsurvey.docx [file mep_2374-8265.11455-s001.zip › H. Circuit Components - Blank.pdf]
